# Single‐Cell Chromatin Accessibility Analysis Reveals Subgroup‐Specific TF‐NTR Regulatory Circuits in Medulloblastoma

**DOI:** 10.1002/advs.202309554

**Published:** 2024-06-17

**Authors:** Xiaoyue Gao, Qiyuan Zhuang, Yun Li, Guochao Li, Zheng Huang, Shenzhi Chen, Shaoxing Sun, Hui Yang, Lan Jiang, Ying Mao

**Affiliations:** ^1^ CAS Key Laboratory of Genome Sciences and Information Beijing Institute of Genomics Chinese Academy of Sciences and China National Center for Bioinformation Beijing 100101 China; ^2^ University of Chinese Academy of Sciences Beijing 100049 China; ^3^ Department of Neurosurgery Huashan Hospital Fudan University Shanghai 200040 China; ^4^ National Center for Neurological Disorders Shanghai Key Laboratory of Brain Function Restoration and Neural Regeneration Neurosurgical Institute of Fudan University Shanghai Clinical Medical Center of Neurosurgery Huashan Hospital Fudan University Shanghai 200040 China; ^5^ Sino‐Danish College University of Chinese Academy of Sciences Beijing 100049 China; ^6^ State Key Laboratory of Medical Neurobiology and MOE Frontiers Center for Brain Science Institute for Translational Brain Research Shanghai Medical College Fudan University Shanghai 200032 China; ^7^ College of Future Technology College University of Chinese Academy of Sciences Beijing 100049 China

**Keywords:** chromatin accessibility landscape, medulloblastoma, neurotransmitter receptors, single‐cell transposase‐accessible chromatin sequencing

## Abstract

Medulloblastoma (MB) stands as one of the prevalent malignant brain tumors among pediatric patients. Despite its prevalence, the intricate interplay between the regulatory program driving malignancy in MB cells and their interactions with the microenvironment remains insufficiently understood. Leveraging the capabilities of single‐cell Assay for Transposase‐Accessible Chromatin sequencing (scATAC‐seq), the chromatin accessibility landscape is unveiled across 59,015 distinct MB cells. This expansive dataset encompasses cells belonging to discrete molecular subgroups, namely SHH, WNT, Group3, and Group4. Within these chromatin accessibility profiles, specific regulatory elements tied to individual subgroups are uncovered, shedding light on the distinct activities of transcription factors (TFs) that likely orchestrate the tumorigenesis process. Moreover, it is found that certain neurotransmitter receptors (NTRs) are subgroup‐specific and can predict MB subgroup classification when combined with their associated transcription factors. Notably, targeting essential NTRs within tumors influences both the in vitro sphere‐forming capability and the in vivo tumorigenic capacity of MB cells. These findings collectively provide fresh insights into comprehending the regulatory networks and cellular dynamics within MBs. Furthermore, the significance of the TF‐NTR regulatory circuits is underscored as prospective biomarkers and viable therapeutic targets.

## Graphical abstract

Medulloblastoma (MB) is a common and serious brain cancer in children. Chromatin accessibility profiles in over 59000 cells are explored from medulloblastoma. Unique regulatory elements are discovered in distinct medulloblastoma subgroups. Certain neurotransmitter receptors, along with specific transcription factors, can predict the tumor subgroups. Targeting these receptors affects tumorigenic capacity and offers new insights into medulloblastoma diagnosis and treatment.

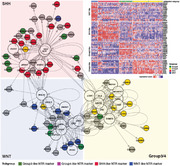

## 1 Introduction

Brain tumors are among the most lethal forms of cancer in children and young adults, due to the unique intrinsic and microenvironmental characteristics of neural tissues that confer resistance to conventional treatments.^[^ ^1^ ^]^ However, the development of targeted therapy methods and the advancement of genomic knowledge have facilitated the identification of specific and efficient therapeutic targets. To this end, the research on heterogeneities among different tumors and within the same tumor subtype is essential. Medulloblastoma (MB), a cerebellar tumor, is one of the most common malignant tumors of the central nervous system in children.^[^ ^2^, ^3^ ^]^ Cancer genomics has revealed four molecular subgroups of medulloblastoma: SHH, WNT, Group3, and Group4, which differ in their genetic, transcriptional, epigenomic, and clinical features.^[^ ^4^, ^5^, ^6^, ^7^, ^8^, ^9^ ^]^ Among these subgroups, only WNT‐MB induces an impaired blood‐brain barrier through paracrine signals, suggesting that the interplay between intrinsic characteristics and tumor microenvironment (TME) is crucial for patient stratification and treatment improvement.^[^ ^10^ ^]^ Moreover, metastatic MB cells require upregulated GABA transaminase to survive in nutrient‐scarce microenvironments, indicating that energy metabolism is another potential therapeutic target.^[^ ^11^ ^]^ Therefore, understanding the internal regulatory program and complex cell‐cell interaction of MB will greatly expand the range of therapeutic approaches.^[^ ^12^ ^]^

Recent studies have applied single‐cell RNA‐seq (scRNA‐seq) to primary MB to reveal the intratumoral heterogeneity and infer the subgroup‐specific developmental origins by comparing MB cell profiles with developing mouse or human cerebellums.^[^ ^13^, ^14^, ^15^, ^16^, ^17^, ^18^, ^19^ ^]^ However, scRNA‐seq is not efficient in measuring the regulatory mechanisms that govern distinct gene expression programs. Bulk measurements of the epigenome have detected enhancers that are differentially regulated and super‐enhancers that contribute to inter‐subgroup heterogeneity and enhancer hijacking in MB.^[^ ^8^, ^20^, ^21^ ^]^ The lack of cellular resolution is unable to uncover the cell‐type‐specific regulatory programs and characterize the tumor microenvironment.

To fill those gaps, here, we performed single‐cell Assay for Transposase‐Accessible Chromatin sequencing (scATAC‐seq) on 11 frozen MB samples of varying ages and mapped the chromatin accessibility landscape of 59015 cells. Our data cover all the major molecular subgroups and reveal the subgroup‐specific *cis*‐regulatory elements (CREs) occupied by different master transcription factors. ScATAC‐seq profile revealed extensive synapse synthesis exists across malignant cells. The expression of selected NTRs and upstream TF regulators from a large cohort helped us develop a new subgroup classification method. Targeting key NTRs in tumors affected both the sphere‐forming ability in vitro and the tumorigenic potential in vivo of MB cells. Overall, our study provides a comprehensive single‐cell regulatory map of four molecular subgroups and reveals the therapeutic vulnerabilities of MB transcription‐factors neurotransmitter receptors regulatory network.

## 2 Result

### 2.1 Single‐Cell Chromatin Landscape of Human MB

**Figure 1:**
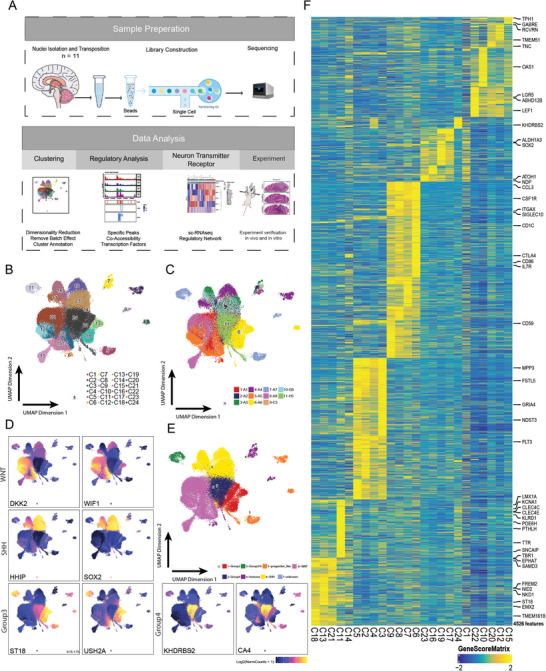
Single‐cell chromatin landscape of human medulloblastoma A) Schematic representation of the samples used in this study (*n* = 11), sequencing experiments, and downstream bioinformatic analyses. B) UMAP of clusters labeled by clusters (*n* = 24). C) UMAP of clusters labeled by samples (*n* = 11). D) Visualization of marker genes of each subgroup. E) UMAP of clusters labeled by subgroups. F) Heatmap of specific gene activity score.

To investigate the chromatin accessibility heterogeneity in MBs, we performed scATAC‐seq (10X Genomics Chromium) on cells of frozen tumor tissues from 11 MB patients (**Figure** **1**A; and Table S1, Supporting Information). There has been no clinical determination of the molecular subgroup of any of the samples. We aimed to assign each sample to a molecular subgroup and to identify the regulatory elements associated with chromatin accessibility.

The raw data was processed to de‐multiplex reads, assign cell barcodes, and align fragments to hg19 by Cell Ranger ATAC. ScATAC‐seq data were filtered based on cut‐offs of 1000 unique nuclear fragments per cell and an enrichment score of 4 for transcription start sites (TSS) in order to eliminate low‐quality data. The filtered cells had an average of 6.2K unique fragments mapping to the nuclear genome (Figure S1A, Supporting Information). In addition, scATAC‐seq profiles show periodic fragment length distributions and enrichment of fragments around TSS (Figure S1A, Supporting Information). Cells passing quality control exhibit a 10.1 median TSS enrichment score and an average of 43.65% of transposition events in all peaks across all the cells (Table S2, Supporting Information). Finally, we removed multiplets by bap2 (≈5%, Figure S1B, Supporting Information).^[^ ^22^ ^]^ All the qualities described above indicated a good performance of scATAC‐seq in frozen human tissues. In total, we obtained 59015 cells after demultiplexing and removing doublets. After that, they were clustered into 24 distinct clusters by ArchR based on chromatin accessibility profiles.^[^ ^23^ ^]^ We then visualized the single‐cell clusters with UMAP (Figure 1B,C,E).

Large‐scale gene expression profiling efforts based on microarrays and scRNA‐seq have characterized genes for molecular subgroups. We performed gene activity scores analysis to infer the gene expression from chromatin accessibility data (Methods). Gene activity scores of each cluster, which are used to classify malignant and immune cells, were calculated from the accessibility of gene windows near the TSS or gene body (Methods). We identified a total of 4526 genes that were uniquely active in each cluster (Figure 1F; and Table S3, Supporting Information). We used a published list of molecular subgroup marker genes, to annotate each cluster and visualize the results on a heatmap and UMAP (Figure 1D).^[^ ^24^ ^]^ All four subgroups (WNT, SHH, Group 3, and Group 4) were observed within the 11 samples. To confirm the annotation, DNA methylation, as the golden standard MB subgrouping method, was profiled. Then 8839 highly variable CpG sites among all the samples was identified for principal component analysis (PCA). Results showed PC1 and PC2 could represent main information of them (Figure S2A, Supporting Information), and the methylation classification based on them is consistent with our scATAC subgroup (Figure S2B, Supporting Information). We further did unsupervised clustering with two PCs (Figure S2C, Supporting Information) or 8839 highly variable CpG sites (Figure S2D, Supporting Information). No matter which dataset was used, the consistent subgroup classification by methylation microarray and scATAC‐seq were observed (Figure S2C,D, Supporting Information).

Then we explore the relationship between four subgroups and 24 scATAC clusters. For example, we detected five clusters (C10, C12, C15, C20, and C22) that expressed *DKK2* and *WIF1*, two key regulators of the WNT pathway. We also observed four clusters (C16, C17, C19, and C23) that showed high activity of *HHIP* and *SOX2*, two markers of the SHH subgroup. Similarly, we identified three clusters (C13, C18, and C21) that had high activity of *ST18* and *USH2A*, which are specific to Group 3, and one cluster (C24) that had high activity of *KHDRBS2* and *CA4*, which are characteristic of Group 4 (Figure 1D). Cluster 11 exhibited a mixed profile of Group 3 and Group 4 genes. Cluster C2‐C5 expressed marker genes in all four subgroups. In addition, the motif enrichment analysis showed that transcript factors are essential for neuronal differentiation (*NEUROD* family), central nervous system development (*NEUROD* family) and ventral neuroectodermal progenitor cell fate (*OLIG2*). Especially, *NEUROD2* is significant in C2‐C5 and several other clusters. However, the binding motif of *NEUROD2* is only active in C2‐C5. Thus, they were annotated as progenitor‐like cells (Figure S1C,D, Supporting Information). Clusters C6‐C9 were annotated as normal cells based on their expression of immune marker genes. Previous literature has reported that macrophages are abundant in the brain tumor microenvironment, especially in the SHH subgroup.^[^ ^25^ ^]^ We found that clusters C6‐C9 had higher accessibility of motifs for *SPI1*, *SPIB*, *JUN* family, and *FOS* family transcription factors, which are involved in myeloid or lymphoid cell development (Figure S1E, Supporting Information). To validate our annotation, we integrated our scATAC‐seq data from C6‐C9 with scRNA‐seq data from a recent MB study.^[^ ^16^ ^]^ We confirmed that all these four clusters had high similarity with macrophages (Figure S1G, Supporting Information).

We examined the contribution of each donor to the immune cell clusters and found that they were largely separated by molecular subgroup (Figure S1F,H, Supporting Information). In other words, C6 was mainly composed of WNT patients, C7 of SHH patients, and C8 and C9 of Group 3 and Group 4 patients. These results indicate that diverse macrophage subpopulations exist in all four MB subgroups and that they have strong subgroup specificity. Immunophenotype may be associated with the subgroup of the patients.

### 2.2 Copy Number Variation Profile of MB

**Figure 2:**
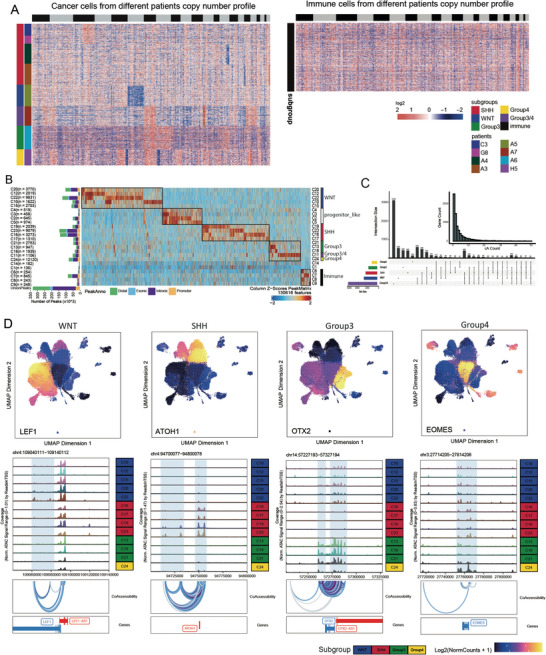
The *cis*‐element regulatory landscape of Medulloblastoma A) CNV patterns of MB cancer (left) and immune (right) cells. B) Peak type distribution of each cluster and heatmap of marker peaks of each cluster. Row means clusters and each column means specific peaks. C) UpSetR plot shows the shared or specific co‐accessible peak target genes among MB subgroups. The barplot in the top right corner indicates the statistics result of genes related to different counts of co‐accessible (cA) peak. D) Peak track of specific co‐accessibility and target genes in each cluster.

Copy number variation (CNV) is an important characteristic to distinguish malignant cells from normal cells and classify tumor cells into different subclones. We computed aneuploid copy number profiles from the ATAC‐seq read counts for each single cell (**Figure** **2**A). The CNV profiles showed that immune cells were very conserved among different patients, which suggests that they can serve as a reference to identify CNV events in tumor cells (Figure 2A). Moreover, we compared the CNV profiles of tumor cells between different patients (Figure 2A; and Figure S3, Supporting Information). We found that some chromosome arm‐level CNV events were specific to different MB subgroups, as reported in previous studies.^[^ ^7^, ^26^ ^]^ For example, both patients A3 and A4 belonged to the SHH subgroup based on our marker gene analysis, and their tumor cells could be clearly distinguished from their immune cells based on their CNV profiles (Figure S3C,D, Supporting Information). Interestingly, tumor cells from patient A3 had chromosomal losses of chr14q and chr17p compared to immune cells, while tumor cells from patient A4 had chromosomal losses of chr17p, which were consistent with the genomic features of SHH (Figure 2A; Figures S3C,D, and S4, Supporting Information).^[^ ^7^, ^26^ ^]^ We also observed distinctive CNV events in tumor cells from different patients, such as chromosomal loss of chr17p in G8 and chromosomal gain of chr3p in both patients G8 and C3 (Figure 2A; Figure S3I,J, Supporting Information). Progenitor‐like tumor cells in patient A4 had a distinct chromosomal loss of chr16p. The CNV profiles of progenitor‐like tumor cells and SHH tumor cells indicated that they belonged to different subclones in patient A4 (Figure S3D, Supporting Information). Patient A5 exhibited WNT cancer cells that were differentiated from other cancer cells, and chromosomal loss of chr6 (also seen in other WNT patients) represents a significant event in the WNT subgroup MB (Figure 2A; Figures S3E and S4, Supporting Information).^[^ ^7^, ^26^ ^]^

Evidence indicates that Group 3/4 cells originate from progenitor‐like cells in patient A7. Group 3/4 cells, for example, have distinct CNV events on chr1q, chr7, chr12p, chr17, chr18 compared with progenitor‐like cells, but other CNV events are consistent with progenitor‐like cells and differ from immune cells, indicating that Group3/4 is a subclone of progenitor cells which also reported as significate mark CNV in Group 3 and Group 4 population (Figure 2A; Figures S3G and S4, Supporting Information).^[^ ^26^ ^]^ These results suggest that CNV analysis from single‐cell ATAC data is reliable for identifying the cell types and related CNV patterns for supporting subgroup annotation.

### 2.3 Cis‐Regulatory Elements Landscape of MB

To characterize the regulatory program of MB, we performed replicate‐aware peak calling on the aggregated pseudo‐bulk profile of annotated clusters. We identified 341426 peaks in total, most of which are intronic (49%) or distal (36%) (Figure 2B; and Table S4, Supporting Information). Furthermore, 38.25% of them (130616) were cluster‐specific peaks (Figure 2B; and Table S5, Supporting Information). Clusters from the same subgroup shared similar specific chromatin accessibility. To elucidate the *cis*‐element regulatory architecture and identify the putative target genes of these *cis*‐regulatory elements (CREs) in specific cell populations, we constructed cis‐co‐accessibility networks (CCANs): modules of sites that are highly co‐accessible with one another separately in each molecular subgroup (Methods).^[^ ^27^ ^]^ For CCANs containing gene promoters, we link each distal CRE to its putative target gene. In total, we identified 38032 CCANs and 7937 target genes across all subgroups, with a median of five cCREs linked to per gene (Figure 2C; and Table S6, Supporting Information). By pairwise comparisons of cCREs target genes identified in each subgroup, we observed a substantial number of genes are shared across multiple subgroups, while some are subgroup‐specific (Figure 2C).

For all subgroups, a significant proportion of subgroup‐specific CCANs containing genes are cell‐type marker genes, and many of them are transcription factors that play key roles in signaling pathways, neurogenesis, and tumorigenesis (Figure S5A, Supporting Information). For example, a CCAN linked to *LEF1*, which encodes the WNT ligand, has been identified specifically in WNT clusters, despite the promoter of *LEF1* being universally open (Figure 2D). CCANs linked to *ATOH1* are identified in SHH clusters, which is consistent with previous reports that *ATOH1^+^* cerebellar granule cell progenitors (GNPs), which express *SOX2* in the developing external granule layer, are particularly vulnerable to SHH‐driven tumorigenesis (Figures 1F and 2D).^[^ ^28^, ^29^ ^]^ Group 3 tumors account for ≈25–30% of MBs and have the worst prognosis. A CCAN linked *OTX2*, whose amplifications are enriched in Group 3 and is identified as super‐enhancer‐associated genes in MBs, exhibits the strongest specificity in Group 3 clusters as well as *HLX* (Figure 2D; Figure S5A,B, Supporting Information).^[^ ^8^ ^]^ *EOMES*, which is a glutamatergic lineage‐specific transcription factor and marks unipolar brush cells (UBCs), is associated with a Group 4 cluster‐specific CCAN, indicating the putative cellular origin of Group 4 (Figure 2D). Interestingly, HOX family genes which relate to embryonic development, morphogenesis, and differentiation, are associated with subgroup‐specific CCAN (Figure S5B, Supporting Information). To confirm the regulatory functions of these cis‐elements, we compare the overlapping of these elements’ region with H3K27ac‐marked super enhancer regions. In this paper, we found 32276 peaks are co‐accessible. Here, we calculated the intersection of co‐accessible peak regions with H3K27ac ChIP‐seq region from frozen modulloblastoma tissue or cell lines and found that 9932 peaks (30.7%) overlap with H3K27ac peaks (Table S7, Supporting Information).^[^ ^8^ ^]^ In addition, two MB cell lines datasets from HACER and cerebellum and fetal brain tissue datasets from EhancerAtlas 2.0 were downloaded and used to do verification.^[^ ^30^, ^31^ ^]^ As a result, 11159 out of 32276 (34.6%) scATAC‐seq peaks were found in at least one dataset (Table S8, Supporting Information), which indicates a relatively large proportion of our peaks have been verified as the functional enhancer in former researches and confirms the reliability of our data.

Altogether, our analyses provide a comprehensive identification of *cis*‐regulatory elements’ activity and reveal the regulatory architecture within different molecular subgroups. Specific peaks, co‐accessible peaks, and CREs’ target genes are identified as putative biomarkers for each subgroup (Tables S5 and S6, Supporting Information). In addition, the genes also revealed the putative regulatory pathway and cellular origin, highlighting the critical role of cCREs for tumorigenesis and subgroup‐specificity determination.

### 2.4 Identification of Specific Regulators in Four Subgroups

**Figure 3:**
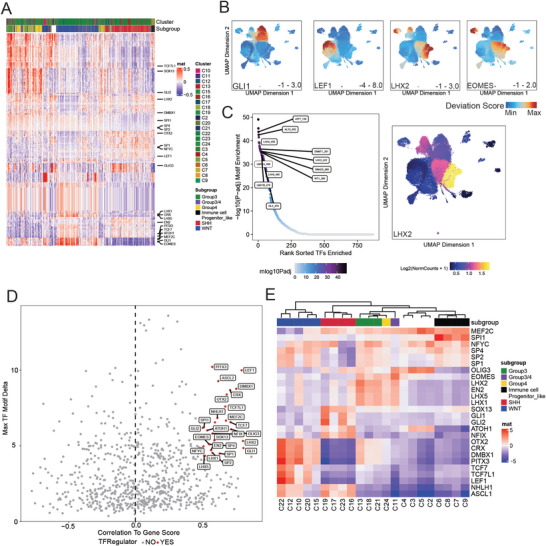
Trans‐regulator in each subgroup. A) Heatmap of active TF motifs accessibility in each cell. Each row indicates each TF, and each column indicates each cell. B) UMAP of the activity of specific putative TF regulators in each subgroup. C) The rank of specific TF regulators in Group 3 (left). The x‐axis means the rank of TFs’ enrichment in this cluster and the y‐axis means motif enrichment score in this cluster. Plots in the left and up mean more specific. And the UMAP of *LHX2’* s gene score(right). D) Positive TF regulators across all of the clusters. X‐axis means the correlation of genes score and motif deviation score, y‐axis means TF motif delta. Labeled TFs are identified as positive regulators. E) Heatmap of positive TF regulator versus Clusters Each row indicates each positive TF regulators in C, and each column indicates each cluster. Each row indicates each positive TF regulators in C, and each column indicates each cluster. Columns were unsupervised clustered based on motif deviation value.

To complement our analysis of *cis*‐elements, we sought to identify cell‐type‐specific trans‐elements regulators. Transcription factors (TF) are important trans elements in regulating gene expression. We constructed a TF network by nominating the putative critical TFs whose binding motifs were co‐enriched in peaks. At the bulk level, we annotated motifs in all peaks using the cisBP database and then constructed a binary matrix where the presence of a motif is indicated numerically in each peak. Then, we examined the motifs that were enriched in marker peaks within each cluster. In addition, we also performed Homer on clusters’ marker peaks to find out cluster‐specific putative TF binding sites enriched in accessible chromatin sites. The putative TFs in each cluster are consistent with significant regulators in known abnormal pathways such as the *GLI* family in SHH, and *LEF1* in WNT (**Figure** **3**A).

We then predict TF activity at the single‐cell level by motif deviation scores from ChromVAR.^[^ ^32^ ^]^ Subgroup‐specific TFs are identified and visualized on UMAP (Figure 3B). And the specific TFs in Group 3 and Group 4 were ranked by enrichment score (Figure 3C). TFs in the same family usually share similar binding motifs, which makes it difficult to distinguish the exact TFs that play a key role in the changes in chromatin accessibility. We then compared the gene score (a proxy for gene expression) and motif deviation score (a proxy for TF binding activity) for each TF across all single cells. The positive correlation of these two scores indicates the positive TF regulatory. After comparing the gene activity scores and motif deviation scores of TFs, we identified 26 positive TF regulators (Figure 3D) and most of them are subgroup‐specific (Figure 3E; Figure S5C, Supporting Information).

Based on the above results, we nominated the cluster‐specific driver TFs (Figure 3D,E). In WNT clusters, *LEF1*, *TCF7L1*, and *TCF7* are positive regulators. In SHH clusters, *GLI1* and *GLI2*, which regulate the SHH pathway, have specific activity. Rational regulatory networks remain scarce for Group 3 and Group 4 subgroup patients. Although they share the most similar TF motifs activity, Group 3 clusters and Group 4 clusters have different gene scores of these genes. The binding motif of *EOMES* is open in both Group 3 and Group 4 clusters, however, the gene only expresses in the Group 4 cluster, which indicates that this transcription factor is only active in Group 4 (Figures 2D and 3B). In Group 3 clusters, *LHX2* shows both high gene scores and high regulatory activity. *LHX2* promotes tumor growth and metastasis, which may explain the frequent metastasis and poor prognosis of Group 3 (Figure 3B,C).^[^ ^33^, ^34^ ^]^

Collectively, our analysis of subgroup‐specific associated TFs and positive TF regulators, not only confirms the previously known regulators for WNT and SHH‐MB but identifies key regulators specifically active in Group 3 and Group 4. Further investigation of *EOMES* and *LHX2* may provide new insight into the etiology and new intervention strategies for Group 3 and Group 4‐MB.

### 2.5 Neurotransmitter Receptor Genes Accurately Predict the Subgroups of MBs

Synaptic communication requires the expression of functional postsynaptic neurotransmitter receptors (NTRs) that match the presynaptically released neurotransmitters (NTs). NTRs are diverse in their types and responses to different NTs, resulting in the diverse response of the post‐synapse cells. For example, glutamate receptors allow sodium ions to pass and excite the post‐synaptic cell, while GABA receptors allow chloride ions to pass and inhibit the post‐synaptic cell. Dysfunction of NTRs has been linked to neuronal system diseases due to their crucial role in neuron communication. In addition, in the GO cellular component enrichment analysis of subgroup marker genes, we also observed the enrichment of neuron‐to‐neuron synapse and synaptic components (Figure S6A, Supporting Information). Therefore, we hypothesized that NTRs are tightly associated with MBs, and that different molecular subgroups may have different types of NTRs.

**Figure 4:**
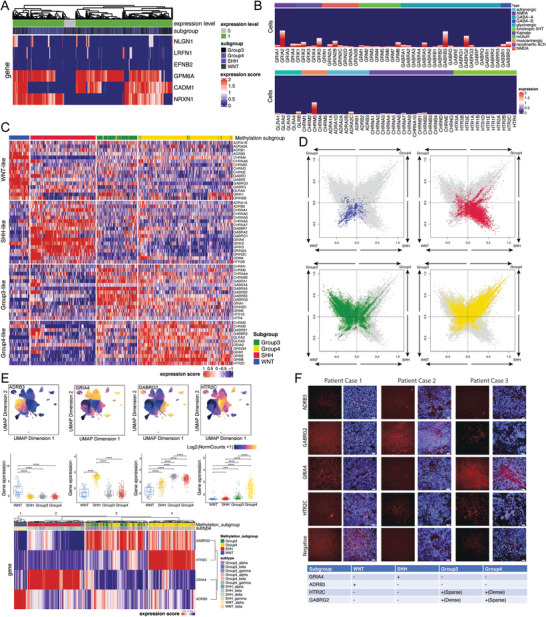
NTR gene specifically expressed in each subtype of medulloblastoma. A) Heatmap of the single‐cell expression level of synaptic synthesis‐related genes in a different subgroup. Each column depicts a single cell from a specific subtype. The color intensity indicates the normalized expression level of each gene in each cell. The top bar shows whether a cell expressed at least one of the five genes that were most strongly associated with synaptogenesis. Most medulloblastoma cells expressed at least one synaptogenic gene, as indicated by the green color. The cells were ordered by hierarchical clustering based on their expression profiles. B) Heatmap with the single‐cell expression of neurotransmitter receptors in the single‐cell medulloblastoma dataset. Each column depicts a single cell. The top annotation indicates receptor types. The x‐axis is ordered by hierarchical clustering, but the dendrogram was omitted for clarity. C) Heatmap of the unsupervised cluster of expression of transmitter receptor‐related genes in medulloblastoma cohort by K‐means (k = 4). Scores are normalized by the Z score. The x‐axis represents the sample (*n* = 763) and the y‐axis represents the gene name. D) 2D representation of single‐cell expression of NTR genes in four subtypes of medulloblastoma. Each quadrant corresponds to one specific subtype, the exact position of malignant cells (dots) reflects their relative scores of four subtypes for the corresponding meta‐modules, and their colors reflect the density of NTR genes. E) Upper panel, UMAP plot of specific NTR gene activity score among clusters. Middle panel, bulk expression level of the NTR genes among groups. Bottom panel, Heatmap with unsupervised clustering of the samples in the cohort (*n* = 763) based on these four genes. Samples were classified by both two subtype classifications. NTR genes have subgroup‐specific chromatin accessibility patterns, indicating differential regulation of these genes among subgroups. F) RNA scope images of each NTR gene marker in representative samples from different subgroups. The pattern was summarized in the bottom table. (Scale bars: 100 µm in right F).

To support the involvement of neurotransmitter receptor genes in MBs’ progression, we examined the expression of NTR genes in two published scRNA‐seq datasets (Data source: GSE119926).^[^ ^13^, ^16^ ^]^ Interestingly, most tumor cells prominently expressed synaptogenesis genes, similar to the reported phenomenon in gliomas.^[^ ^35^, ^36^ ^]^ Synaptic gene enrichment was mainly found in clusters of MBs (**Figure** **4**A,B; Figure S6A, Supporting Information), while various NTRs were upregulated in tumor cells. Furthermore, NTRs showed strong malignant subgroup‐specific expression. By K‐mean unsupervised clustering, the gene expression of 94 reported NTRs was sufficient to classify all 763 MB tumors into several subtypes (Figure 4C,D; Figure S7B,C, Supporting Information). We used unsupervised clustering methods and silhouette plots to determine the optimal cluster number.

Since the expression of some NTR genes was not high in MBs (Figure 4B), we calculated the gene with the highest expression in each group compared to the other groups as the marker gene using one‐way ANOVA. These marker genes classify all 763 MB tumors into 4 subtypes (Figure 4C), which are highly consistent with the golden annotation of MB subgroups by methylation assays and genetic profiles.^[^ ^7^ ^]^ This observation is also supported by the gene activity score calculated from our scATAC data (Figure S6B, Supporting Information). Interestingly, when the unsupervised cluster number was set as 3, the Group 3 and Group 4 were clustered into one group (Figure S7B, Supporting Information). Following this, the Silhouette analysis was used to confirm the stable and optimal cluster number (Figure S7F, Supporting Information). Subsequently, the optimal cluster number was found as 6, which separated SHH and Group 4 into Group A and Group B, respectively (Figure S7C,D, Supporting Information). We further determined clinical characteristics within each subgroup. Specifically, the log‐rank test (*P* < 0.0001) showed six subgroup classifications based on NTR genes of MBs had significant prognosis values (Figure S7E–G, Supporting Information). Finally, we nominated a list of NTR genes as subgroup‐specific biomarkers by combining gene expression data and scATAC data (Figure 4E). We validated these NTR genes by RNAscope in frozen MB samples and confirmed that these NTR genes can identify each subgroup accurately (Figure 4F).

**Figure 5:**
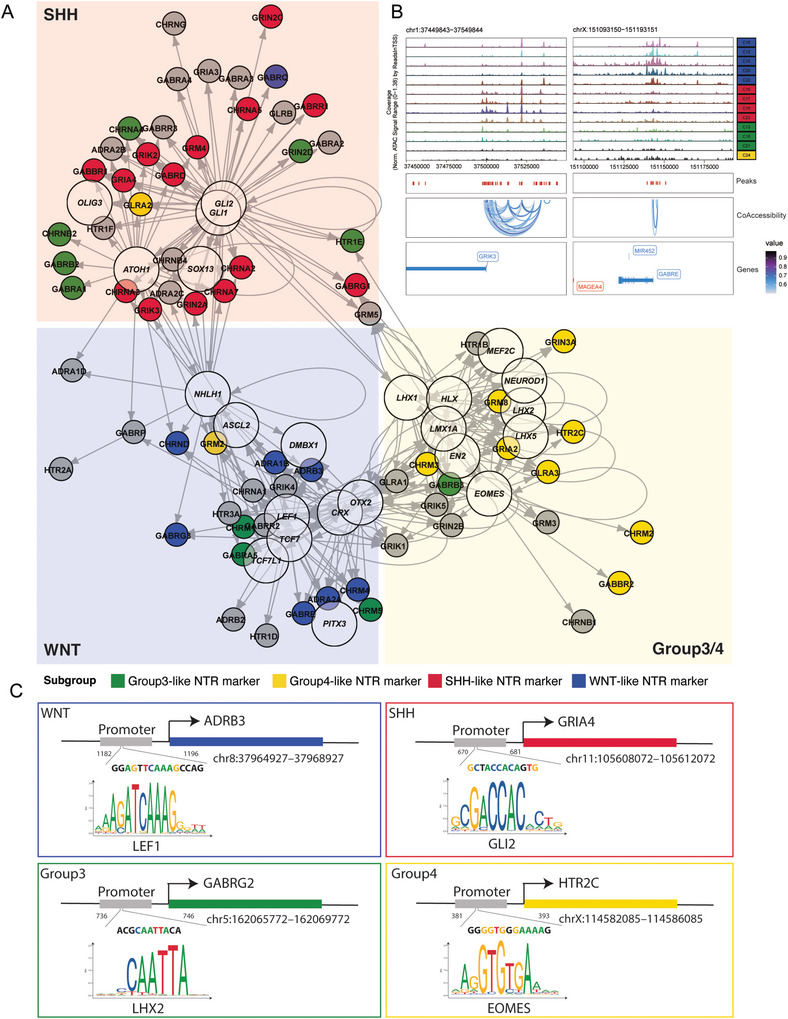
NTR‐TF regulatory network is cluster specific. A) The network figure between TF regulators and NTR genes. Different colors of background indicate subgroup specificity. Bigger and smaller circles indicate TFs and NTR genes, respectively. B) Peak track of co‐accessibility and target NTR genes specific in each cluster. C) Subgroup specific TF binding motifs enrich in promoter of subgroup specific NTR genes.

Our next question was to determine what mechanism underlies the high expression levels and high specificity of these NTR genes in certain subgroups. We observed that there was a significant overlap between the subgroup‐specific CCANs containing the genes mentioned above and the NTR genes. For example, *GRIK3* is an SHH subgroup specifically expressed NTR gene, and a CCAN related to *GRIK3* is identified by only using SHH group scATAC data. The CCAN spans over 50K genomic region and there are dozens of distal ATAC peaks linked to the *GRIK3* promoter. Similarly, a CCAN close to the WNT subgroup specifically expressed NTR gene *GABRE* is found by only using WNT subgroup scATAC‐seq data (**Figure** **5**B). Thus, we conclude that the subgroup specifically regulated NTR gene expression through distal *cis*‐regulatory elements networks.

To identify the upstream regulators of the subgroup‐specific NTR genes, we proposed a trans‐regulatory network for NTR. We took two parallel approaches to search for the upstream regulators of key neuron transmitter receptor genes. In the first step, we calculated the correlation between gene activity scores and TF activity scores across 24 clusters, the correlation indicating a regulating relationship (Table S9, Supporting Information). Second, we annotated the position of motifs across the whole genome, and then identified positive TF motifs that were enriched in peaks in ±5 kb regions of gene transcription beginning sites(Table S10, Supporting Information). These genes were extracted as putative TF target genes. By combining these two gene lists, we constructed a putative NTR‐Positive TF regulatory network (Figure 5A). For each subgroup‐specific positive TF, there are several NTR genes included in their target genes. We then visualized the specific promoter regions of *ADRB3*, *GRIA4*, *GABRG2* and *HTR2C*, respectively, with binding possibility to motif (*LEF1*, *GLI2*, *LHX2*, *EOMES*) (Figure 5C). We then validated the subgroup‐specific TF‐NTR network in Group 3 (HTB‐187), and Group 3/4 cell (HTB‐185) lines (Figure S8A–J, Supporting Information). In Group 3 MB cells, we generated cell lines with LHX2 knockdown and overexpression, while in Group 3/4 MB cells, we created cell lines with EOMES knockdown and overexpression. We found overexpression of EOMES in Group 3/4 MB cells led to increased expression of LHX2 (Figure S8G, Supporting Information, *p* < 0.0001). Similarly, overexpression of LHX2 in Group 3 significantly elevated EOMES expression (Figure S8B, Supporting Information). Knockdown of LHX2 in Group 3 MB cells resulted in decreased GABRG2 expression, whereas overexpression of LHX2 compensated for this decrease in GABRG2 (Figure S8D, Supporting Information). Notably, we did not detect a significant elevation of GABRG2 after LHX2 overexpression, possibly due to the already high baseline expression of GABRG2 in Group 3. Moreover, HTR2C, significantly reduced in Group 3/4 shEOMES #2, exhibited a marked increase after overexpression (Figure S8I, Supporting Information). Furthermore, GRM8, the characteristic NTR of Group 4, showed significant elevation in EOMES overexpression cell lines but not in Group 3 MB cells (Figure S8E,J, Supporting Information). These findings underscore the subgroup‐specific nature of the TF‐NTR network, suggesting the necessity for tailored therapeutic strategies for different medulloblastoma subtypes. In conclusion, we developed a new classification method based on the expression of NTRs and their upstream TF regulators. This method classified MB samples into 4 common subgroups with a limited number of genes. The proteins coded by these genes can not only serve as diagnosis markers but also potentially as therapeutic targets.

### 2.6 NTR Gene Inhibition Suppresses Proliferation and Sphere Formation in MB Cells

**Figure 6:**
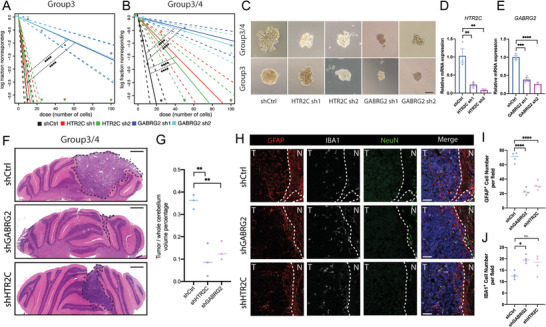
Inhibiting NTRs in MB cells affects the in vitro sphere‐forming ability and in vivo tumorigenic capacity. A,B) Extreme limiting dilution assays (ELDAs) were conducted to assess the ability of cells to form colonies, demonstrating a decrease in neurosphere frequency upon NTR inhibition in Group 3 (HTB‐187) or Group 3/4 (HTB‐185) cells. HTB‐185 and HTB‐187 cells were seeded in 24‐well ultra‐low attachment plates at various concentrations, including 100, 50, 25, 10, and 5 cells per well. Each sphere‐forming well was counted and the dilution ratio was plotted based on the number of diluted cells and the number of sphere‐forming wells. ****P < 0.0001. C) Display of representative spheres derived from Group 3 or Group 3/4 cells expressing either control shRNA (shCtrl), shHTR2C, or shGABRG2. Images of colonies were captured after 1 week of incubation. D,E) Relative *HTR2C* or *GABRG2* expression was measured by qRT‐PCR in shCtrl, shHTR2C, and shGABRG2 Group3/4 cells. Data are shown as the mean ± SEM. **P < 0.01. F) left, HE staining of the maximum cross‐sectional area of G3/4 intracranial orthotopic xenograft model among each group. G) Statistical results of the percentage of medulloblastoma tumor size divide the whole section. Data are expressed as dots. **P < 0.01. H) Immunofluorescence staining of GFAP, IBA1, NeuN revealed less GFAP and increased IBA1 signal in shHTR2C and shGABRG2 groups. Shown are representative images from at least three mice with similar results. I,J) Quantification of GFAP and IBA1 of each group described in H. *P < 0.05, **P < 0.01, ****P < 0.0001. (Scale bars: 50 µm in C, 2 mm in F, 100 µm in H).

As shown in the putative NTR‐Positive TF regulatory network, specific oncogenic pathways in MB subtypes are associated with the aberrant expression of NTR genes. We, therefore, performed subtype‐specific NTR gene knockdown in Group 3, and Group 3/4 cell lines (**Figure** **6**D,E). We assessed the sphere‐forming ability after NTR gene modulation in Group 3 and Group 3/4 MB cells (Figure 6C). Our findings indicated that shHTR2C had a limited impact on Group 3 MB cells, while the knockdown of GABRG2 significantly inhibited the sphere‐forming ability of Group 3 MB cells (Figure 6A,C). Similarly, in Group 3/4 cells, both shHTR2C and shGABRG2 showed a significant effect on the sphere formation ability (Figure 6B,C). We also assessed the sphere‐forming ability after EOMES and LHX2 modulation in Group 3 and Group 3/4 MB cells. Our findings indicated that shLHX2 had a limited impact on sphere formation ability of Group 3 MB cells, while LHX2‐OE showed no statistical significance with shCtrl (Figure S8K,L, Supporting Information). In Group 3/4 cells, shEOMES showed a significant effect on the sphere formation ability, while EOMES‐OE could reverse the inhibition effect (Figure S8K,M, Supporting Information). Furthermore, we noted a modulation of GLI2 expression correlated with changes in EOMES expression levels in Group 3/4 cells when overexpressing EOMES (Figure S8H, Supporting Information). GLI2 serves as a pivotal transcription factor influencing the SHH pathway. Conversely, in the Group 3 cell line, altering EOMES expression did not elicit a change in GLI2 expression (Figure S8C, Supporting Information). This suggests that while EOMES may exert a direct influence on the Group 4 phenotype, it might also interact synergistically with other transcription factors. Thus, NTR genes possibly are more specific subgroup targets.

### 2.7 Decreased Subgroup‐Specific NTR Genes Ameliorate the Growth of Intracranially Implanted MB Tumors

To better simulate clinically relevant conditions, we intracranially implanted shCtrl, shHTR2C, and shGABRG2 Group 3/4 MB cells into the bregma of female nude mice. After 35 days, HE staining revealed that shHTR2C and shGABRG2 led to an amelioration in the growth of intracranially implanted MB tumors compared to shCtrl (Figure 6F,G). Additionally, GFAP is an undifferentiated marker of MB cells. We then examined the GFAP, NeuN, and IBA1 expression around the tumor border. The cell counts of GFAP and IBA1 in different groups demonstrated significantly lower GFAP^+^ cells and increased IBA1^+^ cells in both tumor regions for shHTR2C and shGABRG2 (Figure 6H–J). These results demonstrate that inhibiting subgroup‐specific NTR genes may represent a potential therapeutic target for the treatment of MB.

## 3 Discussion

ScATAC‐seq has advantages in assessing cell‐type differences and developmental and evolutionary dynamics of *cis*‐regulatory elements in cerebellum.^[^ ^37^ ^]^ However, such an analysis has not been reported in primary MBs, especially for long‐term frozen samples due to technical difficulties. In this study, we have developed an optimized protocol to achieve high‐quality data acquisition. Thus, the chromatin accessibility landscape of 11 frozen human MB samples was constructed. Annotation with *cis*‐regulatory elements and gene scores revealed that our samples covered all four MB molecular subgroups. Within these chromatin accessibility profiles, we have uncovered specific regulatory elements tied to individual subgroups, shedding light on the distinct activities of TFs that likely orchestrate the tumorigenesis process.

The transcriptomes of cerebellar tumors reflect similar expression profiles to cell populations in the developing cerebellum, suggesting that the tumors originate from specific cell types that influence their molecular and biological characteristics.^[^ ^17^ ^]^ Previous studies have used transcriptional data to identify biological characteristics and signaling pathways associated with MB subgroups, such as WNT and SHH activation, GABAergic and glutamatergic differentiation, and neuronal lineage specification.^[^ ^13^ ^]^ TFs and chromatin‐associated regulatory complexes integrate signals to RNA polymerase to regulate target gene expression.^[^ ^38^ ^]^ Charles et al. analyzed the enhancers and super‐enhancers that are differentially regulated in MB subgroups and found that they recapitulate and extend the transcriptional and phenotypic features of these subgroups.^[^ ^8^ ^]^ Notably, the core regulatory circuitry constructed in our study drives subgroup‐specific gene expression, uncovering positive TF regulators that were not detected by transcriptional analysis alone. Our approach demonstrates how chromatin accessibility can provide insights into the cellular biological characteristics of tumors.

Neurotransmitters, substances secreted by nerves, modulate neuronal functions by binding to specific receptors.^[^ ^39^ ^]^ Recent studies have revealed the roles of neurotransmitters in regulating the physiology and pathology of various tissues and organs.^[^ ^40^ ^]^ For example, cancer cells exploit the signaling pathway initiated by neurotransmitters to enhance their growth and spread. Moreover, neurotransmitters influence immune and endothelial cells in the tumor microenvironment, facilitating tumor progression.^[^ ^41^ ^]^ Thus, elucidating the mechanisms of neurotransmitter function in cancer development, blood vessel formation, and inflammation may lead to novel antitumor therapies.^[^ ^39^ ^]^ Our GO analysis highlighted the enrichment of neurotransmitter transport and synapse synthesis pathways in malignant cells. The scATAC‐seq profile revealed extensive synapse synthesis exists across malignant cells. Nevertheless, we identified several NTR genes as targets of subgroup‐specific positive TFs. Based on the expression of NTRs and their upstream TF regulators, we developed a new classification method that grouped MB samples into four common subgroups with a limited number of genes. Importantly, we found that targeting key NTRs in tumors affected both the sphere‐forming ability in vitro and the tumorigenic potential in vivo of MB cells. These results reveal new aspects of the regulatory networks and cellular dynamics in MBs. Moreover, we highlight the importance of the TF‐NTR regulatory loops as promising biomarkers and therapeutic targets.

The subgroup classification of MBs is a key issue in the field. Previous studies have identified four main subgroups of MBs based on molecular and clinical features: WNT, SHH, Group 3, and Group 4. Volker et al. reported that Group 3 and Group 4 tumors exhibited a gradient of differentiation from primitive progenitor‐like cells to more mature neuron‐like cells and that the relative proportions of these cell types distinguished these subgroups.^[^ ^13^ ^]^ Florence et al. applied similarity network fusion (SNF) to genome‐wide DNA methylation and gene expression data from 763 MB samples, and found a very homogeneous group of patients that supported the existence of MB subtypes. They also integrated somatic copy number alterations and clinical features to identify 12 distinct MB subtypes within the four main subgroups.^[^ ^42^ ^]^ In this study, we used K‐means unsupervised clustering to analyze the expression of 94 reported NTRs in the same cohort of 763 MB samples. We found that the NTR expression profile was sufficient to classify the samples into four subgroups that were highly consistent with the established MB subgroups by methylation assays and genetic profiles. When we set the cluster number as three, Group 3 and Group 4 samples were merged into one group. We then calculated the accessibility of TF motifs in each subgroup and identified NTR‐specific TF regulators. We also performed scATAC‐seq profiling to reveal positive TF regulators across the cell types within each subgroup. Notably, we found that TF regulators were active only in Group 3 and Group 4 subgroups, which may explain their cellular origin and poor prognosis. Our data suggest an important role of NTRs in determining the cell origin and tumor growth in MBs.

Understanding the cellular origin of different subgroup could lead to the development of improved clinical diagnosis and treatment strategies. Recently, there is considerable evidence that the origins of this disease are related to the development of the normal cerebellum.^[^ ^43^ ^]^ The cells of the cerebellum are derived from two distinct germinal zones. GABAergic neurons, including Purkinje cells, arise from multipotent precursor cells of the primary germinal epithelium in the roof of the fourth ventricular zone. Glutamatergic neurons arise from rhombic lip (RL) region. RL stem cells give rise into two branches cells. GCPs of the external granule layer are generated and then, they proliferate, differentiate, and migrate to become granule neurons of the internal granule layer.^[^ ^44^ ^]^ On the other hand, unipolar brush cells arise.^[^ ^19^ ^]^ In addition, RL splits into RL^VZ^ and RL^SVZ^. RL^VZ^ is most comprised of stem and progenitor cells and cells in the RL^SVZ^ are maturer and migrate into the external granule layer.^[^ ^44^ ^]^ Former researches have revealed that SHH subgroup arises from GCP cells and WNT subgroup arises from cell types surrounding the fourth ventricle.^[^ ^45^ ^]^ *ATOH1*, which is a transcription factor required for GCPs formation and regarded as the marker, expresses in SHH subgroup specifically.^[^ ^46^ ^]^ *OTX2* is expressed in progenitor cells of the RL^SVZ^ zone and *EOMES* is the marker of unipolar brush cells, which are differentially expressed in Group3 and Group4. These results reveal the cellular origin of each subgroup.

Moreover, we found myeloid cells in our samples, as indicated by their signature genes. These cells showed enhanced myeloid cell differentiation, proliferation, and immune response pathways. They also had TF motifs specific to monocytes and macrophages. Previous reports showed SHH MB cells can transdifferentiate into IL‐4‐secreting astrocytes, which induce microglia to release IGF1 and IL‐10, both crucial for tumor progression.^[^ ^47^ ^]^ There is a crosstalk among astrocytes, microglia, and myeloid cells. Moreover, CCL2 (MCP‐1) chemokine and its receptor CCR2, expressed by macrophages, glial, and endothelial cells, promote MB leptomeningeal metastasis.^[^ ^48^ ^]^ In the brain TME, CD163^+^ (M2‐polarised) macrophages/microglia are the main source of CCL2, which recruits CCR4^+^ T‐regs and CCR2^+^ MDSCs.^[^ ^42^ ^]^ We found immune cell clusters were largely contributed by specific molecular subgroup, indicating that diverse macrophage subpopulations exist in four subgroups. Emerging novel immunotherapeutic approaches may regulate the immunosuppressive status of MBs and influence the progression of tumors. Further strategies for immunotherapy should be explored separately according to different categories

In summary, our study provides high resolution of the chromatin accessibility landscape of human MB which can reveal the regulatory network in each MB subgroup. We anticipate that the data and methods presented in our work will be a valuable resource and facilitate the research of regulatory networks, cellular origin, and pathogenic pathways and the development of new treatments for this malignant brain tumor.

## 4 Experimental Section

### Human Patient Tissue Acquisition and Processing

The study of External sample cohorts was added to the 1 ml NbActiv. Human MB tissues were approved by the Reproductive Study Ethics Committee in Huashan Hospital affiliated with Fudan University, China. All tissue samples used for this study were obtained with written informed consent for sample collection and data analyses from all participants. Samples from surgically removed tumor tissues were collected to a cryovial and flash frozen in liquid nitrogen, then transferred to −80 °C for long‐term storage. Eleven individuals ranging from 4 to 40 years old were collected, including 8 males and 3 females (Table S1, Supporting Information).

### Nuclei Isolation

Using forceps, transfer the frozen tissue from the cryovial to a 1.5‐ml microcentrifuge tube. All steps were completed on ice. Tissues were minced into small pieces (≈0.1 mm) on ice using scissors, and then added 500 µl chilled lysis buffer (10 mm Tris‐HCl, pH 7.4, 10 mm NaCl, 3 mm MgCl_2_, 0.01% NP40, 0.01% Tween‐20, and 0.001% digitonin supplemented with 1% BSA), and pipette mix 15 times. After incubation for 5 min on ice, pipetted mix 10 times with a wide‐bore pipette tip. Then, incubated for 10 min on ice before adding 1 ml chilled Wash Buffer (10 mm Tris‐HCl, pH 7.4, 10 mm NaCl, 3 mm MgCl_2_, 0.1% Tween‐20, 1% BSA) to the lysed sample. Pipetted mix 5 times, passed the suspension through a 70 µm Flowmi Cell Strainer before filtered through a 40 µm Flowmi Cell Strainer into a new 1.5 ml tube, then centrifuged at 500 g for 5 min at 4 °C. Removed the supernatant without disrupting the nuclei pellet, then resuspended pellet in chilled Diluted Nuclei Buffer (PN‐2000153, 10x Genomics). If cell debris and large clumps were observed, pass through a 40 µm cell strainer again. For low volume, use a 40 µm Flowmi Cell Strainer (H13680‐0040, Bel‐Art) to minimize volume loss. Determined the nuclei concentration using a Cell Counter.

### ScATAC‐seq Libraries Preparation and Sequencing

The scATAC library was prepared using the 10x Genomics platform with the Chromium Single Cell ATAC Library & Gel Bead Kit (10x Genomics, Pleasanton, California) as instructed by the manufacturer. A total of 15000 nuclei per sample were used as input for single‐cell ATAC‐seq following the manufacturer’ s instructions. Briefly, after tagmentation, the cells were loaded on a Chromium Controller Single‐Cell instrument to generate single‐cell Gel Bead‐In‐Emulsions (GEMs) followed by linear PCR as described in the 10X scATAC‐seq protocol using a Veriti 96‐well thermal cycler (BioRad, 1 851 197). After breaking the GEMs, the barcoded tagmented DNA was purified with SPRIselect Reagent Kit (Beckman Coulter, Pasadena, CA) and further amplified to enable sample indexing and enrichment of scATAC‐seq libraries. The final libraries were quantified using Bioanalyzer (Agilent) and QuBit (Thermofisher) analysis and then sequenced in Nextseq 550AR or NovaSeq 6000 (Illumina, San Diego, CA) with a 50 bp paired‐end read length, or MGISeq‐2000FCL (MGI Tech Co., Ltd., China) with 100‐bp paired‐end read length targeting a depth of 30000 – 50000 reads per cell.

### Whole‐Exome Sequencing

Ten to thirty milligrams tissue from each tumor was used to extract gDNA using the TIANamp Genomic DNA Kit (TIANamp, DP304). DNA was quantitated using the Qubit dsDNA HS Assay Kit with the Qubit 2.0 Fluorometer (Invitrogen, Carlsbad, CA). Three micrograms of each sample genomic DNA were diluted in 1 × TE Buffer (pH 8.0) and sheared to a target peak size of 150 – 200 bp using the Covaris S220 focused‐ultrasonicator (Covaris, Woburn, MA) according to the manufacturer’ s recommendations. Library preparation and exome capture were performed using the SureSelectXT Human All Exon V6 capture baits as described in Agilent's SureSelectXT Target Enrichment System for Illumina Paired‐End Sequencing Library Protocol (version B4) without modification. Eleven cycles of PCR were performed for amplification of the postcapture exome libraries and validated the quality of each library using Agilent's High Sensitivity D1K ScreenTapes on the TapeStation 2200 system.

### Data Processing using Cell Ranger ATAC Software

First, raw Illumina data was demultiplexed into four fastq documents I1, R1, R2, and R3, which included the information of dual index i7 read, read 1, dual index i5 read, and read2, respectively. Then, run the Cell‐ranger count function taking fastq as input. First, the cutadapt tool was used to identify and trim any adapter sequence in each read, and then, BWA was used to align trimmed reads pair to hg19 reference. Finally, fragments and sorted bam files of each sample were got.

### Single‐Cell ATAC‐Seq Data Quality Control

First, ArchR (0.9.5) was used to create arrow files (filterTSS < = 4, filterFrags < = 1000), and then, barcode list in each sample was extracted. Bead‐based scATAC‐seq data Processing (bap) was used to demultiplets. Sorted bam files from Cell Ranger count were input as the origin file, and the barcode list obtained above was input as the barcode‐white list. In this step, several different barcodes of one cell were replaced by one barcode. Then, new fragments, and bam files without multiplets were got. Lastly, new fragments were used as the input of ArchR to create arrow files. In this step, Tile‐Matrix, a sparse cell × bin matrix exhibiting insertion counts across genome‐wide 500‐bp bins, was added to arrow files. Enrichment of ATAC‐seq accessibility at TSSs was used to quantify data quality. Then, ArchR was used to filter the whole scATAC‐seq profile and those had at least 1000 unique fragments and TSS enrichment of 4. Then, the top 12 000 cells of TSS enrichment were kept to avoid overfull cells that would decrease the accuracy of de‐doublet. At last, the doublets enrichment score was calculated by function addDoubletScores() of ArhcR. After filtering doublets, 59015 cells were obtained with a median of 10.01 TSS enrichment and 6.7*10^3^ fragments.

### Dimensionality Reduction, Cell Clustering, and Visualization

ArchR performs Iterative Latent Semantic Indexing (LSI) to reduce dimensionality based on TileMatrix. Default parameters (iterations = 2, dimsToUse = 1:30) were set. Then, Harmony was used to remove the batch effects differences among samples. ArchR uses addClusters() function to add parameters that could be passed to Seurat::FindClusters() function, implementing a deterministic clustering result. In this step, 24 clusters were identified. Then, the visualization by UMAP was finished based on Iterative LSI and harmony dimensionality reduction matrix, respectively. Default parameters (nNeighbors = 30, minDist = 0.5) were set.

### Inferring Copy Number Profile by scATAC‐seq Data

To infer DNA copy number profiles from scATAC‐seq data, a method previously described was adapted. This method estimates CNVs by determining read counts in 10 Mb windows with 2 Mb shifting across the genome and comparing read counts in each window with the average read count in 100 GC‐matched intervals, which have the most similar 100 GC content values. By this method, it would have system false‐positive copy number signals, due to the unbalanced distribution of GC contents across the whole genome. To overcome this problem, two modifications were made. First, false‐positive values always appeared in windows with low CG content values. So, low CG content value windows were filtered according to the distribution of CG content value of windows. Second, matched normal cells were used as a reference to reduce false‐postive CNV events. To achieve this, the genome was first tiled into 10‐Mb windows using “slidingWindows” of GenomicRanges for chromosome sizes in R with a step size of 2 Mb. These window positions were then filtered with known artifactual mapping issues using the ENCODE hg19 blacklist with the “setdiff” function in R. After that low CG content windows would be filtered, a threshold was selected according to the correlation between CG content values windows and false‐positive copy number signals windows. Then cell‐by‐window binarized matrix was constructed, as described above. The percentage GC nucleotide content was computed for each filtered 10‐Mb window using the hg19 BSgenome in R. To single‐cell infer copy number signals, the 100 nearest neighbors were identified based on GC content and computed the average log2(fold change). Then these values were used to minus matched average reference cells log2(fold change). If it was above 1, this region was considered to be a candidate for amplification. In contrast, this region was a candidate for deletion. This approach was previously validated in with matched whole exome sequencing data from an earlier study in two patient samples.^[^ ^49^ ^]^ Indeed, CNVs identified using scATAC‐seq could be confirmed by matched whole exome sequencing in these two patients (A7 and H5) samples.

### Peak Calling and Motif Identification

ArchR offered addReproduciblePeakSet() function for peak calling by Macs2. After peak calling, marker peaks in each cluster were obtained by getMarkerFeatures() using PeakMatrix. Then, motif information obtained from CisBP was added to annotate the motif region's overlap peaks.

### Transcription Factors Activity Identification

ChromVAR was used to predict the enrichment of TF activity on a per‐cell basis from sparse chromatin accessibility data. MotifMatrix including deviations (how different the motif in one cell deviates from others) and z‐scores (the normalization of deviations) was added based on motif annotation of peaks.

### Gene Activity Score Calculation

First, the chromosome was divided into 500 bp tiles, and then, these tiles were overlapped with the gene window (100 kb on either side of the transcription start site of the gene). After that, the distance from each tile to the gene body was converted to a distance weight. Second, the inverse of gene size (1/gene size) was applied to remove the bias of gene size on gene score. Third, the number of Tn5 insertions in the tile was summed. At last, the gene score was got after multiplying the three elements described above and summing across all tiles in the gene window. Gene activity scores could reflect gene expression.

### Annotation of Major Cell Types

ScATAC‐seq subpopulations for WNT, SHH, Group3, and Group4 were annotated based on gene activity score, using canonical marker genes. Subgroup‐specific marker genes were revealed by gene expression profiles. Immune cells were annotated using Seurat label‐transfer prediction scores with the scRNA‐seq clusters as a reference annotation. The R package ComplexHeatmap was used for hierarchical clustering and visualization of these gene activity matrices.

### Confirm MB Subgrouping by DNA Methylation

To confirm the annotation by scATAC‐seq data, DNA methylation was profiled by Illumina Infinium MethylationEPIC v2.0 BeadChip as the gold‐standard MB subgrouping method. Then the “ENmix” R package was used for analysis, including quality control, background correction, dye bias correction, between‐array normalization, probe‐type bias adjustment, filtering rows and columns with too many missing values, and imputation. A methylation level (beta values) matrix of 935355 CpG sites * 11 samples were obtained after the above data‐preprocessing. Then 8839 highly variable CpG sites (standard deviation among all samples > 0.3) were selected for analysis. Then the MB subgrouping result by CpG sites methylation pattern was compared with that by scATAC‐seq data, with principal component analysis and unsupervised clustering (“pheatmap” R package, default clustering parameters).

### TF Regulatory Network

Using scATAC‐seq in one cell type, candidate TF regulatory target genes were identified and used this information and gene activity score to construct cell‐type‐specific TF regulatory networks. The same set of TF binding motifs were used as those used in the single‐cell TF motif enrichment analysis (Cisbp). For a given TF, the correlation of motif deviation scores and gene activity scores between each TF to all of the genes were first calculated and a correlation score >0.5 was set as a cut‐off. Then, candidate target genes were defined as those with an accessible promoter or distal regions containing the TF binding motif. This information was used to construct a directed TF regulatory network using the R package igraph, where each vertex represents a TF or target gene, and each edge represents a putative regulate event.

### Chromatin CCANs Analysis

The correlation structure of chromatin accessibility data was analyzed using the addCoAccessibility() function in ArchR.

### Prediction of NTR's Transcription Factors

The correlation between gene activity score and motif deviation score was calculated. Genes (23 274) were paired to 870 motifs and 20248380 pairs of correlation were calculated in the total. Correlation >0.5 and padj <0.01 were set as the cutoff. TF binding motifs were classified across the whole genome by the addMotifAnnotations() function in ArchR. Then, ATAC Peaks were mapped to TF binding motifs by bedtools intersect. Then, motifs enriched in promoter and distal regions of interested genes were selected.

### Cell Culture

All cell lines were maintained at 37 °C in humidified incubators with 5% CO_2_ from ATCC (ATCC HTB‐185, HTB‐187). According to the instruction of ATCC, HTB‐187 were cultured in EMEM (ATCC‐formulated Eagle’ s Minimum Essential Medium, Catalog No. 30–2003) containing 20% fetal bovine serum (Gibco BRL, USA) and 1% streptomycin (Gibco BRL, USA). HTB‐185 was cultured in EMEM containing 10% FBS and 1% streptomycin. For stable cell line construction, cells were infected by condensed lentivirus. Cells were transient transfected with plasmids by LipofectamineTM 2000 Transfection Reagent (Invitrogen) according to the manufacturer's instructions.

### RNA Extraction and Real‐Time Quantitative PCR (RT–qPCR)

RNA was extracted according to the manufacturer's instructions of the EZ‐press RNA purification kit (B0004D, EZBioscience, USA), and following quantified by UV spectrophotometer. Extracted RNA was transcribed into cDNA reversely and amplified by EZ‐press Cell to cDNA Kit (B0003, EZBioscience, USA). QuantStudio 6 Real‐Time PCR system (Applied Biosystems, Foster City, CA, USA) and SYBR Green qPCR Master Mix (Vazyme Biotech, Nanjing, China) were used to perform qPCR. The primers used were as follows: GRM8 sense strand: 5′‐CGAGGGAAAGCGATCAGCC‐3′, antisense strand: 5′‐CCCATCCACCCGTATGGAA; GLI2 sense strand: 5′‐CCCCTACCGATTGACATGCG‐3′, antisense strand: 5′‐GAAAGCCGGATCAAGGAGATG; EOMES sense strand: 5′‐GCCATGCTTAGTGACACCGA‐3′, antisense strand: 5′‐GGACTGGAGGTAGTACCGC; HTR2C sense strand: 5′‐CTAGTGGGACTACTTGTCATGCC‐3′, antisense strand: 5′‐GCGATATAGCGCAGAGGTGCAT; LHX2 sense strand: 5′‐ATGCTGTTCCACAGTCTGTCG‐3′, antisense strand: 5′‐GCATGGTCGTCTCGGTGTC; GABRG2 sense strand: 5′‐ACTTCGGCCTGATATAGGAGTG‐3′, antisense strand: 5′‐

ACGTCTGTCATACCACGTTTG. All qPCR were repeated three times. one‐way ANOVA tests and subsequent Dunnett tests were used for comparisons within multiple groups. *P* < 0.05 was considered statistically significant. GraphPad Prism 8 were used for statistical analysis.

### Sphere‐Formation Assay and Extreme Limiting Dilution Assay

For HTB‐185 and HTB‐187 cells, a range of cell concentrations (100, 50, 25, 10, 5 cells per well) were plated in 24‐well ultra‐low attachment plates. The cells were allowed to form spheres for 7–10 days or until fully formed spheres were observed in the control group. The number of spheres in each well was recorded. MB‐spheres were visualized and imaged using an inverted phase‐contrast microscope. Extreme Limiting Dilution analysis was performed by http://bioinf.wehi.edu.au/software/elda. All experiments were independently repeated at least three times.

### HE Staining and Immunofluorescence

Mouse brains were collected and fixed by perfusion with PBS and 4% paraformaldehyde. The brains were then subjected to deparaffinization and rehydration to obtain frozen sections of 10 µm thickness. Hematoxylin and eosin (H&E) staining was carried out using standard procedures. For immunofluorescence staining, a blocking solution containing normal sheep serum (5%) was applied to the sections. Subsequently, the sections were incubated with primary antibodies (GFAP at 1:100, No. OB‐PRT001; NeuN at 1:100 No. OB‐PRT045; and IBA1 at 1:300, No. NB100‐1028) at 4 °C overnight. Following three washes with TBS, the sections were exposed to secondary antibodies for 1 h at room temperature. Finally, the images were captured using a Zeiss camera. This methodology enabled the visualization and analysis of specific markers in the brain tissue sections, aiding in the characterization of cellular components and their spatial distribution.

### Group 3/4 Intracranial Orthotopic Xenograft Model

The mice were precisely positioned using a stereotaxic head frame and anesthesia mask (Stereotaxis for mouse, 68 055 Adaptor) to ensure accurate injection. Surgical anesthesia was induced using a mixture of 2% isoflurane and oxygen. Subsequently, 1 × 10^6^ shHTR2C, shGABRG2, and shCtrl Group 3/4 cells in 2 µl PBS were carefully injected into the cerebellum of 6–8week‐old female nude mice (BABL/c, Gempharmatech Co., Ltd, China) at the coordinates 2 mm lateral to midline and 1 mm posterior to lambda. After 35 days, the mice were harvested for subsequent tumor staining. All animal experiments were performed following the approved protocols of the Department of Laboratory Animal Science, ensuring ethical compliance, and animal welfare.

### RNA Scope

RNA scope experiments were performed following the manufacturer's instructions (Cat. No. 323 100). In brief, the RNAscope Multiplex Fluorescent Detection Reagents were employed to assess the expression of *ADRB3*, *HTR2C*, *GRIA4*, and *GABRG2* genes in human MB FFPE sections. After deparaffinization and antigen retrieval, the sections were subjected to RNAscope ISH using specific probes for each target gene. Fluorescent images were acquired using a fluorescence microscope, facilitating the precise and simultaneous localization of *ADRB3*, *HTR2C*, *GRIA4*, and *GABRG2* gene expression within the MB sections.

### Statistical Analysis

Statistical analysis was performed by Prism 8 software or commercially available software (SPSS 22.0). The results were expressed as mean ± standard error of means (SEMs). Differences between groups were evaluated using one‐way analysis of variance (ANOVA). A significance level of *P* < 0.05 was considered statistically significant.

### Ethics Statement

The use of human tissues (KY2021‐458) was approved by the Ethics Committee of Huashan Hospital affiliated to Fudan University, China. The informed consent of all participating subjects was obtained. All animal studies strictly adhered to approved protocols by the Ethics Committee of the Shanghai Medical School (202311006Z). All procedures were carried out according to the approved guidelines.

## Conflict of Interest

The authors declare no conflict of interest.

## Author Contributions

X.G., Q.Z., Y.L., and G.L. contributed equally to this work as co‐first authors. Y.M., L.J., and H.Y. designed and supervised this study. Y.M., H.Y., and Q.Z. collected the patients’ samples and clinical information. Y.L. performed sample process and constructed the library. X.G., G.L., S.C., Z.H., and S.S. analyzed the scATAC‐seq data and scRNA‐seq data, Y.M., H.Y., and Q.Z. analyzed the cohort data and found that NTR genes can be used for classifying MB. L.J., X.G., Q.Z., and S.C. wrote the original draft.

## Supporting information

Supporting Information

Supplemental Table 1

Supplemental Table 2

Supplemental Table 3

Supplemental Table 4

Supplemental Table 5

Supplemental Table 6

Supplemental Table 7

Supplemental Table 8

Supplemental Table 9

Supplemental Table 10

## Data Availability

The data that support the findings of this study are available from the corresponding author upon reasonable request.

## References

[1] K. Aldape , K. M. Brindle , L. Chesler , R. Chopra , A. Gajjar , M. R. Gilbert , N. Gottardo , D. H. Gutmann , D. Hargrave , E. C. Holland , D. T. W. Jones , J. A. Joyce , P. Kearns , M. W. Kieran , I. K. Mellinghoff , M. Merchant , S. M. Pfister , S. M. Pollard , V. Ramaswamy , J. N. Rich , G. W. Robinson , D. H. Rowitch , J. H. Sampson , M. D. Taylor , P. Workman , R. J. Gilbertson , Nat. Rev. Clin. Oncol. 2019, 16, 509. 30733593 10.1038/s41571-019-0177-5 PMC6650350

[2] P. A. Northcott , G. W. Robinson , C. P. Kratz , D. J. Mabbott , S. L. Pomeroy , S. C. Clifford , S. Rutkowski , D. W. Ellison , D. Malkin , M. D. Taylor , A. Gajjar , S. M. Pfister , Nat. Rev. Dis. Primers 2019, 5, 11. 30765705 10.1038/s41572-019-0063-6

[3] P. A. Northcott , A. Korshunov , S. M. Pfister , M. D. Taylor , Nat. Rev. Neurol. 2012, 8, 340. 22565209 10.1038/nrneurol.2012.78

[4] M. Remke , T. Hielscher , P. A. Northcott , H. Witt , M. Ryzhova , A. Wittmann , A. Benner , A. von Deimling , W. Scheurlen , A. Perry , S. Croul , A. E. Kulozik , P. Lichter , M. D. Taylor , S. M. Pfister , A. Korshunov , J. Clin. Oncol. 2011, 29, 2717. 21632505 10.1200/JCO.2011.34.9373

[5] M. D. Taylor , P. A. Northcott , A. Korshunov , M. Remke , Y. J. Cho , S. C. Clifford , C. G. Eberhart , D. W. Parsons , S. Rutkowski , A. Gajjar , D. W. Ellison , P. Lichter , R. J. Gilbertson , S. L. Pomeroy , M. Kool , S. M. Pfister , Acta Neuropathol. 2012, 123, 465. 22134537 10.1007/s00401-011-0922-z PMC3306779

[6] P. A. Northcott , I. Buchhalter , A. S. Morrissy , V. Hovestadt , J. Weischenfeldt , T. Ehrenberger , S. Grö;bner , M. Segura‐Wang , T. Zichner , V. A. Rudneva , H. J. Warnatz , N. Sidiropoulos , A. H. Phillips , S. Schumacher , K. Kleinheinz , S. M. Waszak , S. Erkek , D. T. W. Jones , B. C. Worst , M. Kool , M. Zapatka , N. Jä;ger , L. Chavez , B. Hutter , M. Bieg , N. Paramasivam , M. Heinold , Z. Gu , N. Ishaque , C. Jäger‐Schmidt , et al., Nature 2017, 547, 311. 28726821 10.1038/nature22973 PMC5905700

[7] F. M. G. Cavalli , M. Remke , L. Rampasek , J. Peacock , D. J. H. Shih , B. Luu , L. Garzia , J. Torchia , C. Nor , A. S. Morrissy , S. Agnihotri , Y. Y. Thompson , C. M. Kuzan‐Fischer , H. Farooq , K. Isaev , C. Daniels , B. K. Cho , S. K. Kim , K. C. Wang , J. Y. Lee , W. A. Grajkowska , M. Perek‐Polnik , A. Vasiljevic , C. Faure‐Conter , A. Jouvet , C. Giannini , A. A. Nageswara Rao , K. K. W. Li , H. K. Ng , C. G. Eberhart , et al., Cancer Cell 2017, 31, 737. 28609654 10.1016/j.ccell.2017.05.005 PMC6163053

[8] C. Y. Lin , S. Erkek , Y. Tong , L. Yin , A. J. Federation , M. Zapatka , P. Haldipur , D. Kawauchi , T. Risch , H. J. Warnatz , B. C. Worst , B. Ju , B. A. Orr , R. Zeid , D. R. Polaski , M. Segura‐Wang , S. M. Waszak , D. T. Jones , M. Kool , V. Hovestadt , I. Buchhalter , L. Sieber , P. Johann , L. Chavez , S. Gröschel , M. Ryzhova , A. Korshunov , W. Chen , V. V. Chizhikov , K. J. Millen , et al., Nature 2016, 530, 57. 26814967 10.1038/nature16546 PMC5168934

[9] D. N. Louis , A. Perry , P. Wesseling , D. J. Brat , I. A. Cree , D. Figarella‐Branger , C. Hawkins , H. K. Ng , S. M. Pfister , G. Reifenberger , R. Soffietti , A. von Deimling , D. W. Ellison , Neuro Oncol 2021, 23, 1231. 34185076 10.1093/neuonc/noab106 PMC8328013

[10] T. N. Phoenix , D. M. Patmore , S. Boop , N. Boulos , M. O. Jacus , Y. T. Patel , M. F. Roussel , D. Finkelstein , L. Goumnerova , S. Perreault , E. Wadhwa , Y. J. Cho , C. F. Stewart , R. J. Gilbertson , Cancer Cell 2016, 29, 508. 27050100 10.1016/j.ccell.2016.03.002 PMC4829447

[11] V. Martirosian , K. Deshpande , H. Zhou , K. Shen , K. Smith , P. Northcott , M. Lin , V. Stepanosyan , D. Das , J. Remsik , D. Isakov , A. Boire , H. De Feyter , K. Hurth , S. Li , J. Wiemels , B. Nakamura , L. Shao , C. Danilov , T. Chen , J. Neman , Cell Rep. 2021, 36, 109475. 34320362 10.1016/j.celrep.2021.109475 PMC12776182

[12] D. F. Quail , J. A. Joyce , Cancer Cell 2017, 31, 326. 28292436 10.1016/j.ccell.2017.02.009 PMC5424263

[13] V. Hovestadt , K. S. Smith , L. Bihannic , M. G. Filbin , M. L. Shaw , A. Baumgartner , J. C. DeWitt , A. Groves , L. Mayr , H. R. Weisman , A. R. Richman , M. E. Shore , L. Goumnerova , C. Rosencrance , R. A. Carter , T. N. Phoenix , J. L. Hadley , Y. Tong , J. Houston , R. A. Ashmun , M. DeCuypere , T. Sharma , D. Flasch , A. Silkov , K. L. Ligon , S. L. Pomeroy , M. N. Rivera , O. Rozenblatt‐Rosen , J. M. Rusert , R. J. Wechsler‐Reya , et al., Nature 2019, 572, 74. 31341285 10.1038/s41586-019-1434-6 PMC6754173

[14] V. Hovestadt , O. Ayrault , F. J. Swartling , G. W. Robinson , S. M. Pfister , P. A. Northcott , Nat. Rev. Cancer 2020, 20, 42. 31819232 10.1038/s41568-019-0223-8 PMC9113832

[15] L. N. Gonzalez Castro , I. Liu , M. Filbin , Neuro Oncol 2023, 25, 234. 36197833 10.1093/neuonc/noac211 PMC9925698

[16] K. A. Riemondy , S. Venkataraman , N. Willard , A. Nellan , B. Sanford , A. M. Griesinger , V. Amani , S. Mitra , T. C. Hankinson , M. H. Handler , M. Sill , J. Ocasio , S. J. Weir , D. S. Malawsky , T. R. Gershon , A. Garancher , R. J. Wechsler‐Reya , J. R. Hesselberth , N. K. Foreman , A. M. Donson , R. Vibhakar , Neuro Oncol 2022, 24, 273. 34077540 10.1093/neuonc/noab135 PMC8804892

[17] M. C. Vladoiu , I. El‐Hamamy , L. K. Donovan , H. Farooq , B. L. Holgado , Y. Sundaravadanam , V. Ramaswamy , L. D. Hendrikse , S. Kumar , S. C. Mack , J. J. Y. Lee , V. Fong , K. Juraschka , D. Przelicki , A. Michealraj , P. Skowron , B. Luu , H. Suzuki , A. S. Morrissy , F. M. G. Cavalli , L. Garzia , C. Daniels , X. Wu , M. A. Qazi , S. K. Singh , J. A. Chan , M. A. Marra , D. Malkin , P. Dirks , L. Heisler , et al., Nature 2019, 572, 67. 31043743 10.1038/s41586-019-1158-7 PMC6675628

[18] L. D. Hendrikse , P. Haldipur , O. Saulnier , J. Millman , A. H. Sjoboen , A. W. Erickson , W. Ong , V. Gordon , L. Coudière‐Morrison , A. L. Mercier , M. Shokouhian , R. A. Suárez , M. Ly , S. Borlase , D. S. Scott , M. C. Vladoiu , H. Farooq , O. Sirbu , T. Nakashima , S. Nambu , Y. Funakoshi , A. Bahcheli , J. J. Diaz‐Mejia , J. Golser , K. Bach , T. Phuong‐Bao , P. Skowron , E. Y. Wang , S. A. Kumar , P. Balin , et al., Nature 2022, 609, 1021. 36131014 10.1038/s41586-022-05215-w PMC10026724

[19] K. S. Smith , L. Bihannic , B. L. Gudenas , P. Haldipur , R. Tao , Q. Gao , Y. Li , K. A. Aldinger , I. Y. Iskusnykh , V. V. Chizhikov , M. Scoggins , S. Zhang , A. Edwards , M. Deng , I. A. Glass , L. M. Overman , J. Millman , A. H. Sjoboen , J. Hadley , J. Golser , K. Mankad , H. Sheppard , A. Onar‐Thomas , A. Gajjar , G. W. Robinson , V. Hovestadt , B. A. Orr , Z. Patay , K. J. Millen , P. A. Northcott , Nature 2022, 609, 1012. 36131015 10.1038/s41586-022-05208-9 PMC9748853

[20] M. R. Corces , J. M. Granja , S. Shams , B. H. Louie , J. A. Seoane , W. Zhou , T. C. Silva , C. Groeneveld , C. K. Wong , S. W. Cho , A. T. Satpathy , M. R. Mumbach , K. A. Hoadley , A. G. Robertson , N. C. Sheffield , I. Felau , M. A. A. Castro , B. P. Berman , L. M. Staudt , J. C. Zenklusen , P. W. Laird , C. Curtis , W. J. Greenleaf , H. Y. Chang , C. G. A. A. Network , Science 2018, 362.

[21] P. A. Northcott , C. Lee , T. Zichner , A. M. Stütz , S. Erkek , D. Kawauchi , D. J. Shih , V. Hovestadt , M. Zapatka , D. Sturm , D. T. Jones , M. Kool , M. Remke , F. M. Cavalli , S. Zuyderduyn , G. D. Bader , S. VandenBerg , L. A. Esparza , M. Ryzhova , W. Wang , A. Wittmann , S. Stark , L. Sieber , H. Seker‐Cin , L. Linke , F. Kratochwil , N. Jäger , I. Buchhalter , C. D. Imbusch , G. Zipprich , et al., Nature 2014, 511, 428. 25043047 10.1038/nature13379 PMC4201514

[22] C. A. Lareau , S. Ma , F. M. Duarte , J. D. Buenrostro , Nat. Commun. 2020, 11, 866. 32054859 10.1038/s41467-020-14667-5 PMC7018801

[23] J. M. Granja , M. R. Corces , S. E. Pierce , S. T. Bagdatli , H. Choudhry , H. Y. Chang , W. J. Greenleaf , Nat. Genet. 2021, 53, 403. 33633365 10.1038/s41588-021-00790-6 PMC8012210

[24] P. A. Northcott , A. Korshunov , H. Witt , T. Hielscher , C. G. Eberhart , S. Mack , E. Bouffet , S. C. Clifford , C. E. Hawkins , P. French , J. T. Rutka , S. Pfister , M. D. Taylor , J. Clin. Oncol. 2011, 29, 1408. 20823417 10.1200/JCO.2009.27.4324 PMC4874239

[25] A. S. Margol , N. J. Robison , J. Gnanachandran , L. T. Hung , R. J. Kennedy , M. Vali , G. Dhall , J. L. Finlay , A. Erdreich‐Epstein , M. D. Krieger , R. Drissi , M. Fouladi , F. H. Gilles , A. R. Judkins , R. Sposto , S. Asgharzadeh , Clin. Cancer Res. 2015, 21, 1457. 25344580 10.1158/1078-0432.CCR-14-1144 PMC7654723

[26] P. A. Northcott , D. J. Shih , J. Peacock , L. Garzia , A. S. Morrissy , T. Zichner , A. M. Stütz , A. Korshunov , J. Reimand , S. E. Schumacher , R. Beroukhim , D. W. Ellison , C. R. Marshall , A. C. Lionel , S. Mack , A. Dubuc , Y. Yao , V. Ramaswamy , B. Luu , A. Rolider , F. M. Cavalli , X. Wang , M. Remke , X. Wu , R. Y. Chiu , A. Chu , E. Chuah , R. D. Corbett , G. R. Hoad , S. D. Jackman , et al., Nature 2012, 488, 49. 22832581 10.1038/nature11327 PMC3683624

[27] H. A. Pliner , J. S. Packer , J. L. McFaline‐Figueroa , D. A. Cusanovich , R. M. Daza , D. Aghamirzaie , S. Srivatsan , X. Qiu , D. Jackson , A. Minkina , A. C. Adey , F. J. Steemers , J. Shendure , C. Trapnell , Mol. Cell 2018, 71, 858. 30078726 10.1016/j.molcel.2018.06.044 PMC6582963

[28] D. S. Malawsky , S. J. Weir , J. K. Ocasio , B. Babcock , T. Dismuke , A. H. Cleveland , A. M. Donson , R. Vibhakar , K. Wilhelmsen , T. R. Gershon , Commun Biol 2021, 4, 616. 34021242 10.1038/s42003-021-02099-w PMC8139976

[29] H. J. Selvadurai , E. Luis , K. Desai , X. Lan , M. C. Vladoiu , O. Whitley , C. Galvin , R. J. Vanner , L. Lee , H. Whetstone , M. Kushida , T. Nowakowski , P. Diamandis , C. Hawkins , G. Bader , A. Kriegstein , M. D. Taylor , P. B. Dirks , Cell Rep. 2020, 31, 107511. 32294450 10.1016/j.celrep.2020.03.075

[30] J. Wang , X. Dai , L. D. Berry , J. D. Cogan , Q. Liu , Y. Shyr , Nucleic Acids Res. 2019, 47, D106. 30247654 10.1093/nar/gky864 PMC6323890

[31] T. Gao , J. Qian , Nucleic Acids Res. 2020, 48, D58. 31740966 10.1093/nar/gkz980 PMC7145677

[32] A. N. Schep , B. Wu , J. D. Buenrostro , W. J. Greenleaf , Nat. Methods 2017, 14, 975. 28825706 10.1038/nmeth.4401 PMC5623146

[33] A. Kuzmanov , U. Hopfer , P. Marti , N. Meyer‐Schaller , M. Yilmaz , G. Christofori , Mol. Oncol. 2014, 8, 401. 24423492 10.1016/j.molonc.2013.12.009 PMC5528541

[34] F. Zhou , S. Gou , J. Xiong , H. Wu , C. Wang , T. Liu , Mol. Biol. Rep. 2014, 41, 8163. 25324171 10.1007/s11033-014-3716-2

[35] V. Venkataramani , D. I. Tanev , C. Strahle , A. Studier‐Fischer , L. Fankhauser , T. Kessler , C. Körber , M. Kardorff , M. Ratliff , R. Xie , H. Horstmann , M. Messer , S. P. Paik , J. Knabbe , F. Sahm , F. T. Kurz , A. A. Acikgöz , F. Herrmannsdörfer , A. Agarwal , D. E. Bergles , A. Chalmers , H. Miletic , S. Turcan , C. Mawrin , D. Hänggi , H. K. Liu , W. Wick , F. Winkler , T. Kuner , Nature 2019, 573, 532. 31534219 10.1038/s41586-019-1564-x

[36] H. S. Venkatesh , W. Morishita , A. C. Geraghty , D. Silverbush , S. M. Gillespie , M. Arzt , L. T. Tam , C. Espenel , A. Ponnuswami , L. Ni , P. J. Woo , K. R. Taylor , A. Agarwal , A. Regev , D. Brang , H. Vogel , S. Hervey‐Jumper , D. E. Bergles , M. L. Suvà , R. C. Malenka , M. Monje , Nature 2019, 573, 539. 31534222 10.1038/s41586-019-1563-y PMC7038898

[37] I. Sarropoulos , M. Sepp , R. Frömel , K. Leiss , N. Trost , E. Leushkin , K. Okonechnikov , P. Joshi , P. Giere , L. M. Kutscher , M. Cardoso‐Moreira , S. M. Pfister , H. Kaessmann , Science 2021, 373. 34446581 10.1126/science.abg4696 PMC7611596

[38] D. Shlyueva , G. Stampfel , A. Stark , Nat. Rev. Genet. 2014, 15, 272. 24614317 10.1038/nrg3682

[39] S. H. Jiang , L. P. Hu , X. Wang , J. Li , Z. G. Zhang , Oncogene 2020, 39, 503. 31527667 10.1038/s41388-019-1006-0

[40] R. Mittal , L. H. Debs , A. P. Patel , D. Nguyen , K. Patel , G. O'Connor , M. Grati , J. Mittal , D. Yan , A. A. Eshraghi , S. K. Deo , S. Daunert , X. Z. Liu , J. Cell. Physiol. 2017, 232, 2359. 27512962 10.1002/jcp.25518 PMC5772764

[41] M. Mancino , E. Ametller , P. Gascón , V. Almendro , Biochim. Biophys. Acta 2011, 1816, 105. 21616127 10.1016/j.bbcan.2011.04.005

[42] A. L. Chang , J. Miska , D. A. Wainwright , M. Dey , C. V. Rivetta , D. Yu , D. Kanojia , K. C. Pituch , J. Qiao , P. Pytel , Y. Han , M. Wu , L. Zhang , C. M. Horbinski , A. U. Ahmed , M. S. Lesniak , Cancer Res. 2016, 76, 5671. 27530322 10.1158/0008-5472.CAN-16-0144 PMC5050119

[43] R. J. Gilbertson , D. W. Ellison , Annu Rev Pathol 2008, 3, 341. 18039127 10.1146/annurev.pathmechdis.3.121806.151518

[44] P. Haldipur , K. A. Aldinger , S. Bernardo , M. Deng , A. E. Timms , L. M. Overman , C. Winter , S. N. Lisgo , F. Razavi , E. Silvestri , L. Manganaro , H. Adle‐Biassette , F. Guimiot , R. Russo , D. Kidron , P. R. Hof , D. Gerrelli , S. J. Lindsay , W. B. Dobyns , I. A. Glass , P. Alexandre , K. J. Millen , Science 2019, 366, 454. 31624095 10.1126/science.aax7526 PMC6897295

[45] P. Gibson , Y. Tong , G. Robinson , M. C. Thompson , D. S. Currle , C. Eden , T. A. Kranenburg , T. Hogg , H. Poppleton , J. Martin , D. Finkelstein , S. Pounds , A. Weiss , Z. Patay , M. Scoggins , R. Ogg , Y. Pei , Z. J. Yang , S. Brun , Y. Lee , F. Zindy , J. C. Lindsey , M. M. Taketo , F. A. Boop , R. A. Sanford , A. Gajjar , S. C. Clifford , M. F. Roussel , P. J. McKinnon , D. H. Gutmann , et al., Nature 2010, 468, 1095. 21150899 10.1038/nature09587 PMC3059767

[46] C. H. Chang , M. Zanini , H. Shirvani , J. S. Cheng , H. Yu , C. H. Feng , A. L. Mercier , S. Y. Hung , A. Forget , C. H. Wang , S. M. Cigna , I. L. Lu , W. Y. Chen , S. Leboucher , W. J. Wang , M. Ruat , N. Spassky , J. W. Tsai , O. Ayrault , Dev. Cell 2019, 48, 184. 30695697 10.1016/j.devcel.2018.12.017

[47] M. Yao , P. B. Ventura , Y. Jiang , F. J. Rodriguez , L. Wang , J. S. A. Perry , Y. Yang , K. Wahl , R. B. Crittenden , M. L. Bennett , L. Qi , C. C. Gong , X. N. Li , B. A. Barres , T. P. Bender , K. S. Ravichandran , K. A. Janes , C. G. Eberhart , H. Zong , Cell 2020, 180, 502. 31983537 10.1016/j.cell.2019.12.024 PMC7259679

[48] L. Garzia , N. Kijima , A. S. Morrissy , P. De Antonellis , A. Guerreiro‐Stucklin , B. L. Holgado , X. Wu , X. Wang , M. Parsons , K. Zayne , A. Manno , C. Kuzan‐Fischer , C. Nor , L. K. Donovan , J. Liu , L. Qin , A. Garancher , K. W. Liu , S. Mansouri , B. Luu , Y. Y. Thompson , V. Ramaswamy , J. Peacock , H. Farooq , P. Skowron , D. J. H. Shih , A. Li , S. Ensan , C. S. Robbins , M. Cybulsky , et al., Cell 2018, 173, 1549. 10.1016/j.cell.2018.05.033 29856958

[49] K. E. Yost , A. T. Satpathy , D. K. Wells , Y. Qi , C. Wang , R. Kageyama , K. L. McNamara , J. M. Granja , K. Y. Sarin , R. A. Brown , R. K. Gupta , C. Curtis , S. L. Bucktrout , M. M. Davis , A. L. S. Chang , H. Y. Chang , Nat. Med. 2019, 25, 1251. 31359002 10.1038/s41591-019-0522-3 PMC6689255

