# Supplementary material for: Single‐Cell Chromatin Accessibility Analysis Reveals Subgroup‐Specific TF‐NTR Regulatory Circuits in Medulloblastoma
Source: Adv Sci (Weinh). 2024 Jun 17;11(30):2309554. doi: 10.1002/advs.202309554 (PMC11321678; doi:10.1002/advs.202309554)
Supplement: Supplementary file 1 — Supporting Information [file ADVS-11-2309554-s006.docx]

**Supporting Information**

**Single-cell chromatin accessibility analysis reveals subgroup-specific TF-NTR regulatory circuits in medulloblastoma**

*Xiaoyue Gao, Qiyuan Zhuang, Yun Li, Guochao Li, Zheng Huang, Shenzhi Chen, Shaoxing Sun, Hui Yang,** *Lan Jiang,** *Ying Mao**


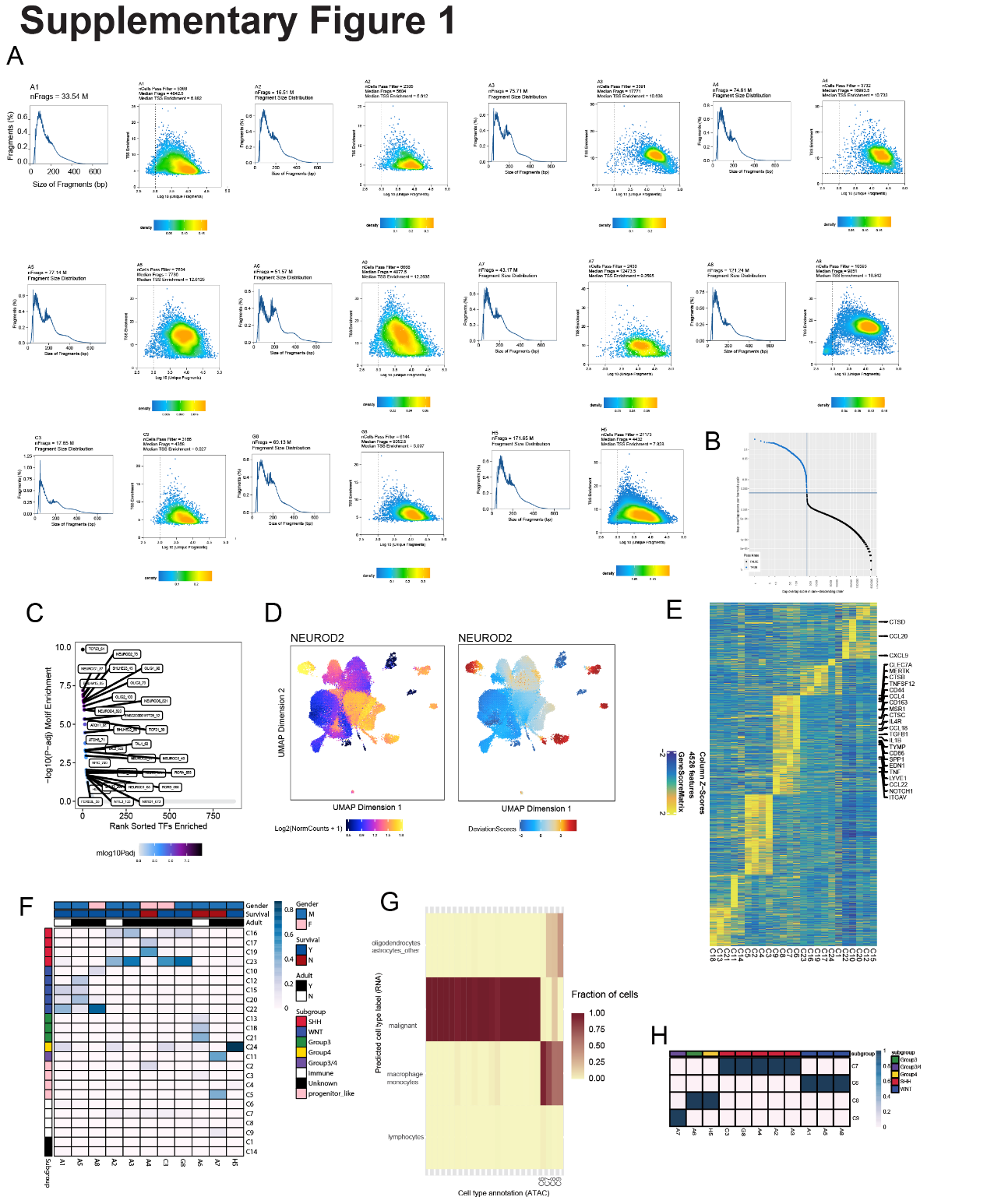


**Figure S1. Data quality and sample information.** A) Fragments size distribution(left) and TSS enrichment and cells passing filter(right) for each sample(n=11). B) bap2 doublets remove. Y-axis indicates bap overlap score per barcode pair. X-axis indicates bap overlap score in ranking-descending order. C) TF binding motifs enriched in cluster 3. X-axis indicates sorted rank of enriched TFs. Lower rank indicates stronger enrichment. Y-axis means -log10(P-adj) of the binding motif. Higher score indicates more significant. D) Visualization of gene score (left) or motif deviation score (right) of NEUROD2. Higher score indicates higher expression and accessibility. E) Heatmap of gene activity score in each cluster. Labeled genes are immune genes. F) Cluster distribution in each sample. Each column indicates each sample, each row indicates cluster. G) Heatmap of immune cluster annotation. By integrating former scRNA-seq data with our scATAC-seq data to predict the cell type of each cluster. Column indicates cluster and row indicates predicted cell type. H) Heatmap of distribution of immune clusters in each sample.


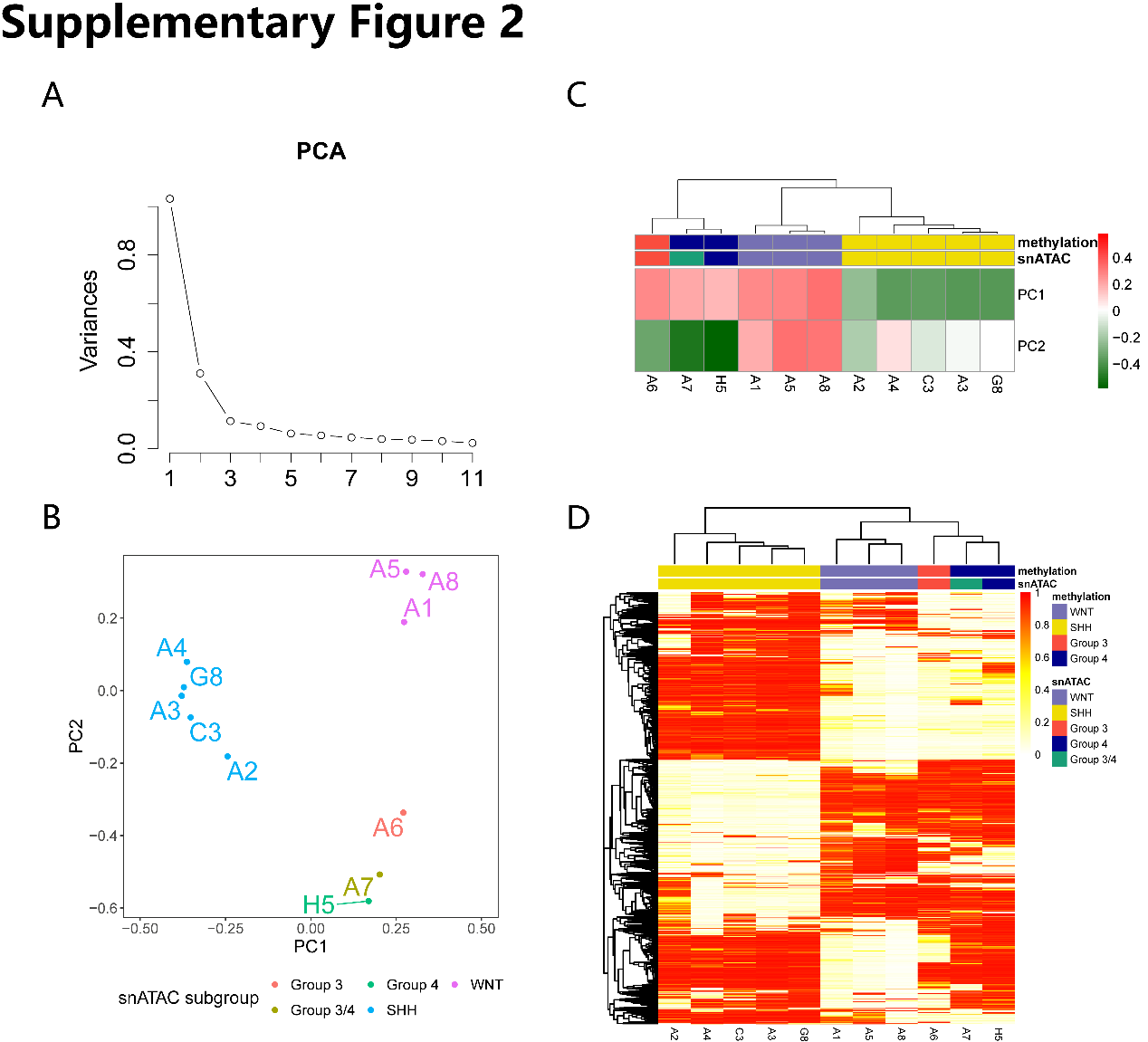


**Figure S2. Comparison of subgroup classification by methylation microarray and that by snATAC-seq data.** A) PCA scree plot of 8839 highly variable CpG sites (sd > 0.4) from methylation microarray. B) The samples in the same snATAC subgroup have the similar methylation PC1 and PC2. C) Unsupervised clustering with two methylation PCs showed the same subgroup classification with that based on snATAC data. D) Unsupervised clustering with 8839 highly variable CpG sites showed the same subgroup classification with that based on snATAC data.


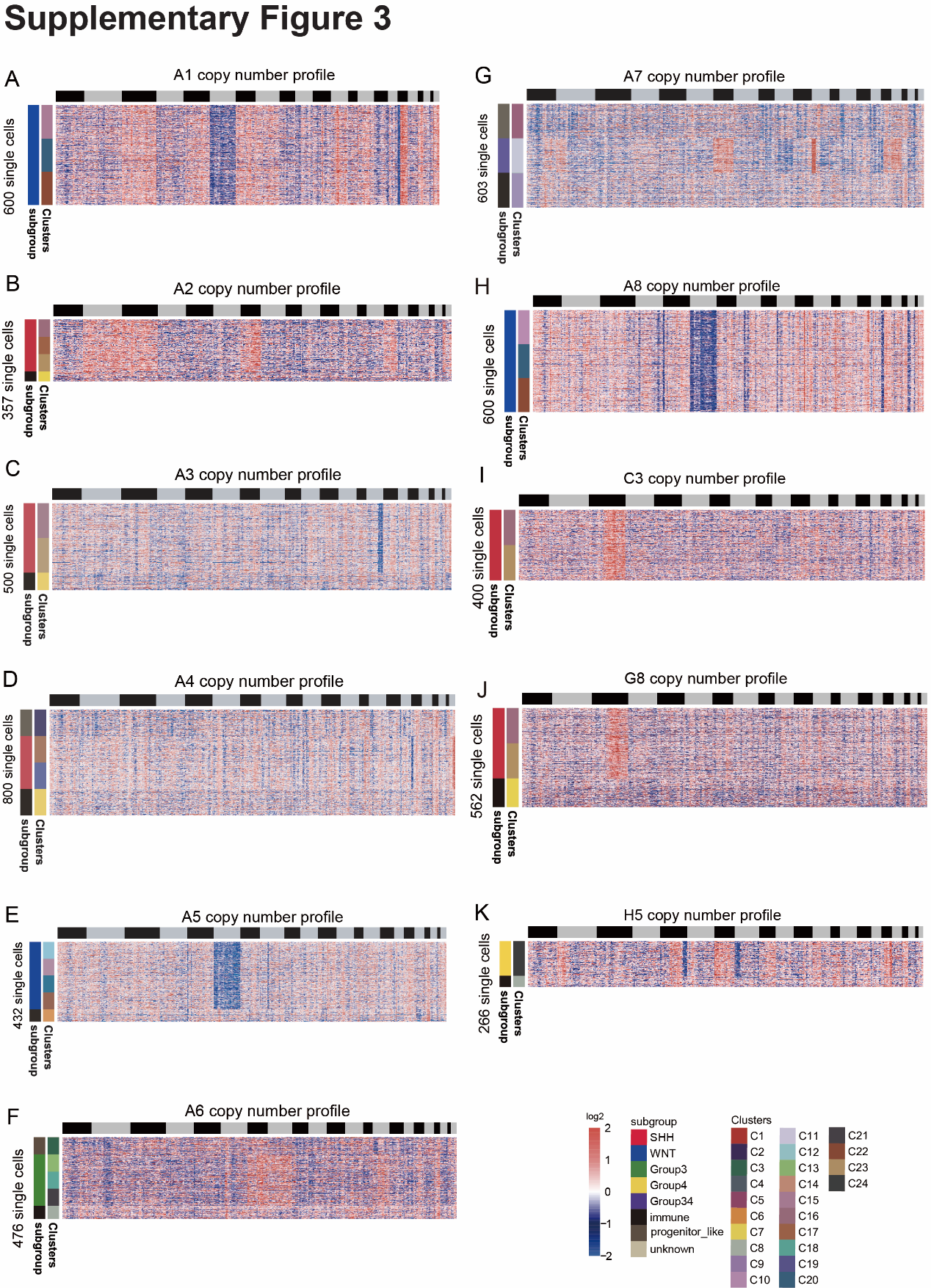


**Figure S3. CNV pattern.** A-K. CNV patterns in different samples. Annotation bars on the left of each heat map indicate subgroups and clusters.


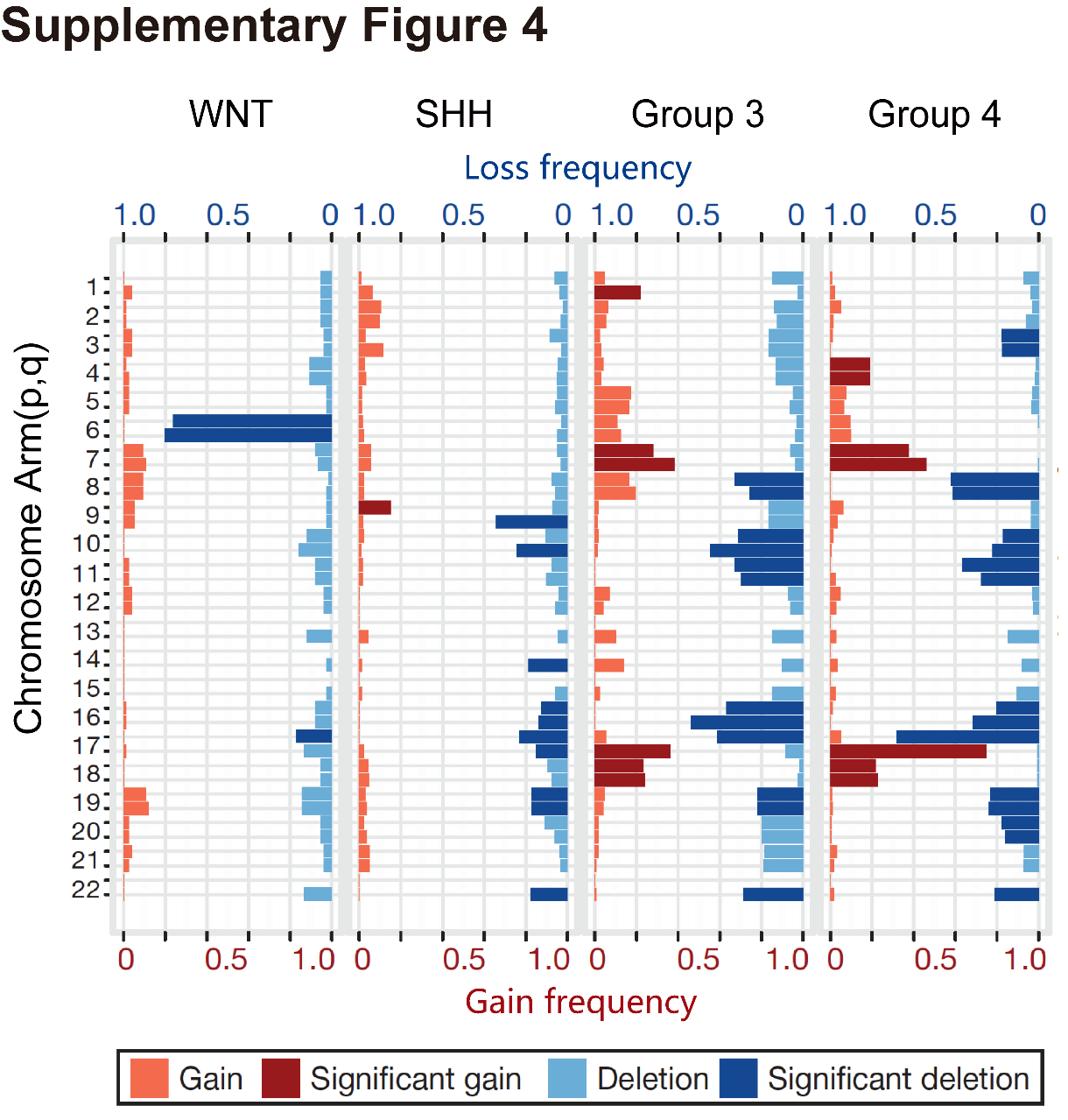


**Figure S4. Frequency and significance (Q value ≤ 0.1) of broad cytogenetic events (chromosome lost and gain) across medulloblastoma subgroup populations.** This figure is cited from reference [1].


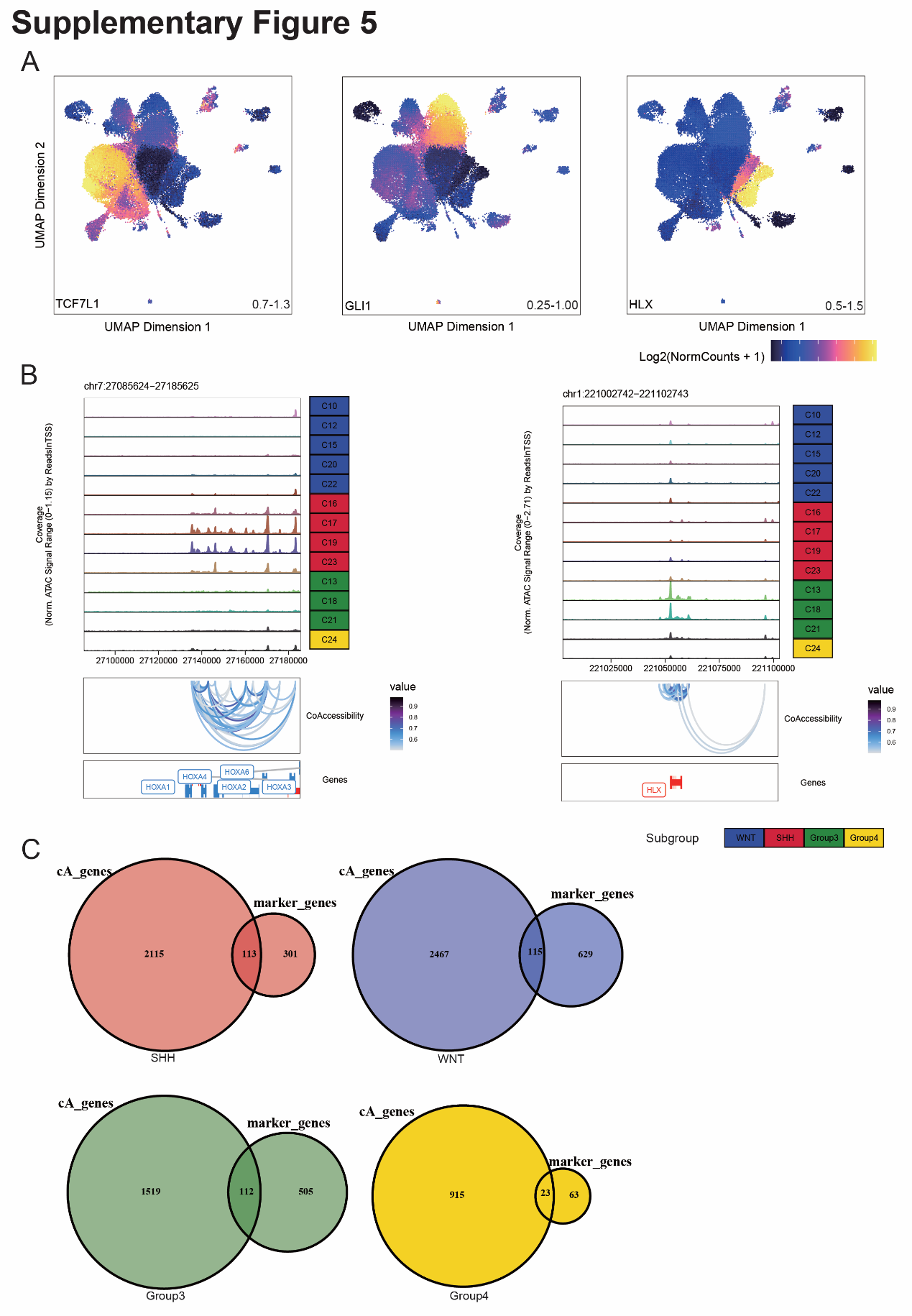


**Figure S5. Co-accessibility and target genes.** A) UMAP of subgroup specific genes in each cluster. Yellow color indicates higher gene score. B) Co-accessibility (cA) track and related genes in SHH (left) and G3 (right) subgroups. C) Overlap of cA related genes and specific expressed marker genes in each subgroup.


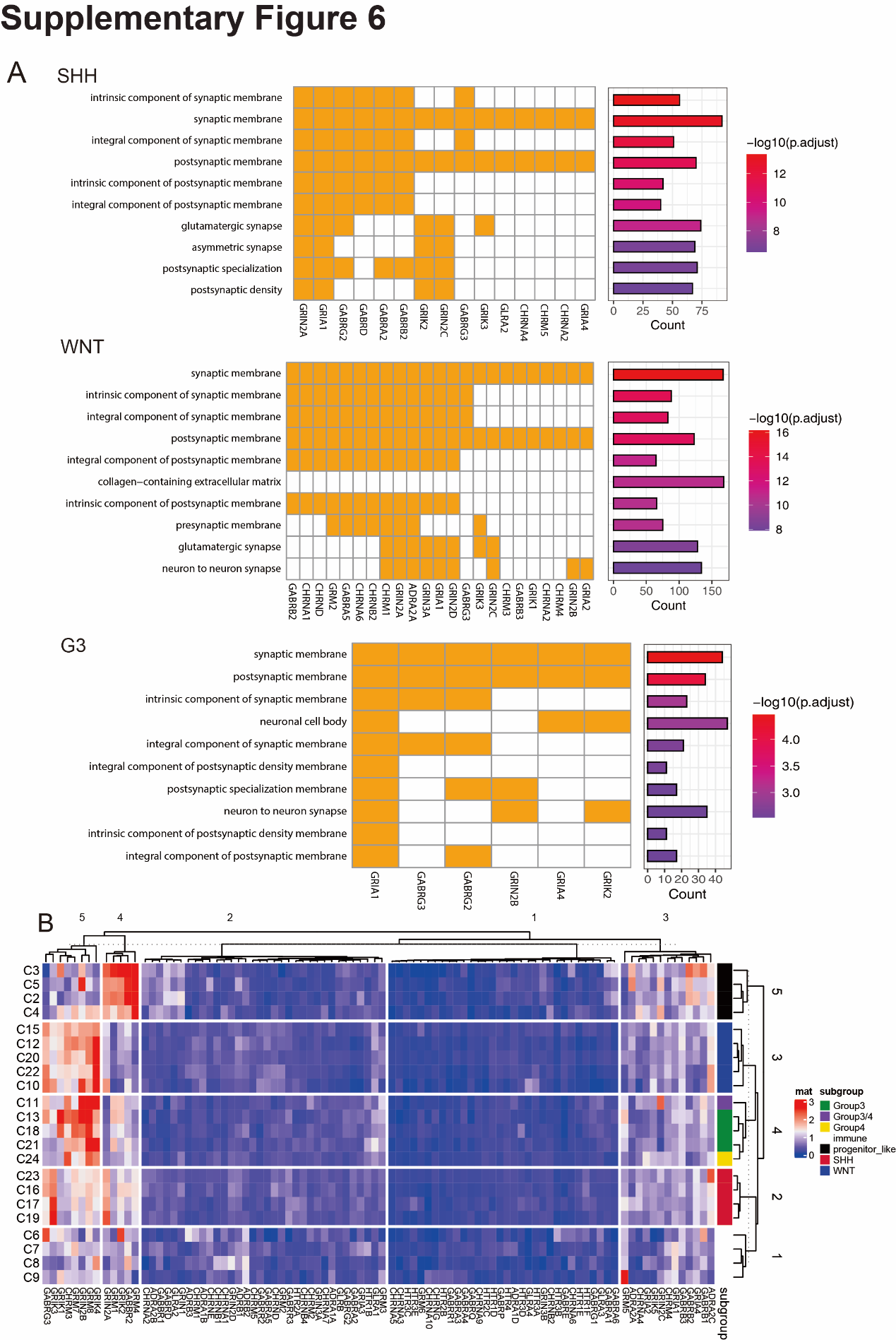


**Figure S6. Neurotransmitter receptors in MB.** A) Top 10 GO cellular component enrichment terms in each subgroup. NTR related pathways were observed. B) Heatmap of NTR genes in each cluster. Clusters were clustered based on NTR gene score.


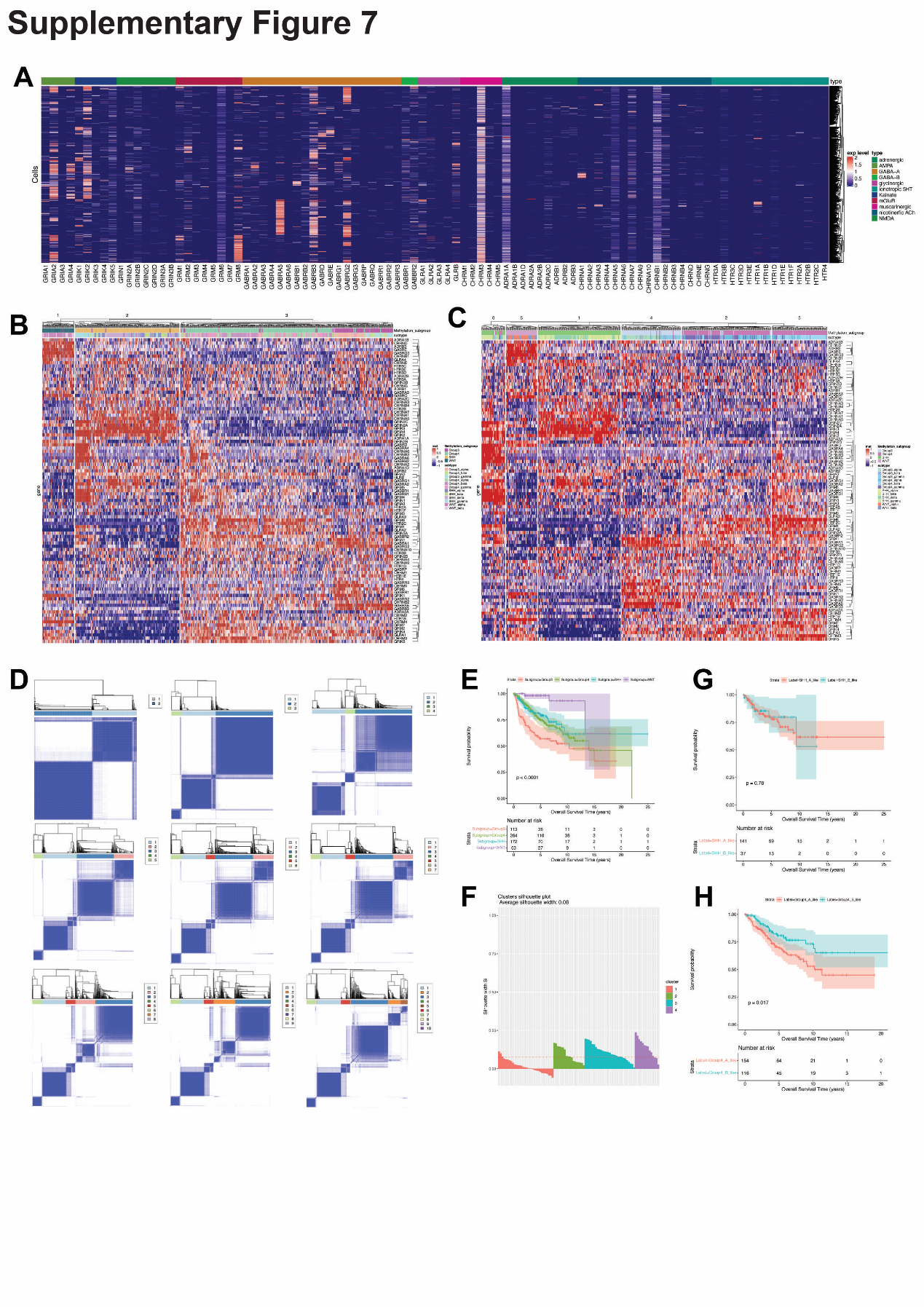


**Figure S7. Specific expression of the NTR gene is of great interest for classification of medulloblastoma.** A) Heatmap with the single-cell expression of neurotransmitter receptors in the single-cell medulloblastoma dataset. Each column depicts a single cell. The top annotation indicates receptor types. The y-axis is ordered by hierarchical clustering. B-C) Heatmaps of the unsupervised cluster of expression of transmitter receptor-related genes in medulloblastoma cohort by K-means (k = 3 or 6). Scores are normalized by the Z score. The x-axis represents the sample (n = 763) and the y-axis represents the gene name. D) k-means unsupervised clustering of expression of transmitter receptor-related genes in medulloblastoma cohort (k number set from 2-10). E) Kaplan-Meier plot of four NTR groups (k number = 4) in medulloblastoma cohort. The P-value was from Kaplan–Meier log-rank test. F) Silhouette analysis of clustering result (k = 4). G-H) Kaplan-Meier plot of different groups classified by NTR groups (k number = 6) in medulloblastoma cohort, respectively. The P-value was from Kaplan–Meier log-rank test.


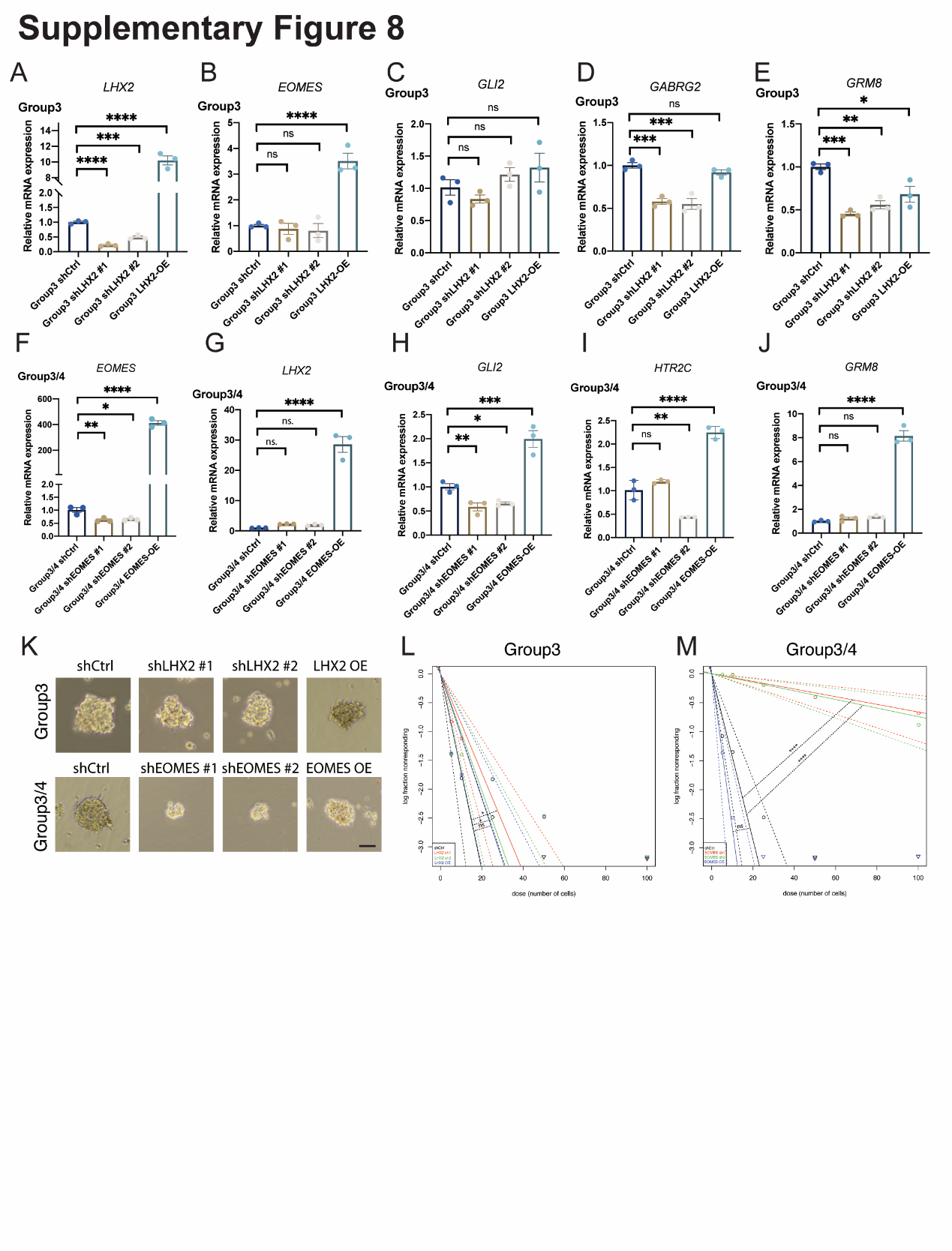


**Figure S8. Regulate specific TFs affect subgroup NTR expression alteration and sphere formation.** A-E) Relative TFs and subgroup specific NTR expression were measured by qRT-PCR among shCtrl, shLHX2 and LHX2-OE Group 3 cells. Data are shown as the mean ± SEM. one-way ANOVA tests and subsequent Dunnett tests were used for comparisons within multiple groups. *P <0.05, **P <0.01, ***P <0.001, ****P <0.0001, ns. P>0.05. F-J) Relative TFs and subgroup specific NTR expression were measured by qRT-PCR among shCtrl, shEOMES and EOMES-OE Group 3/4 cells. Data are shown as the mean ± SEM. one-way ANOVA tests and subsequent Dunnett tests were used for comparisons within multiple groups. *P <0.05, **P <0.01, ***P <0.001, ****P <0.0001, ns. P>0.05. K) Display of representative spheres derived from Group 3 or Group 3/4 cells expressing either control shRNA (shCtrl), shLHX2, shEOMES, LHX2-OE and EOMES-OE. Images of colonies were captured after 1 week of incubation. L-M) Extreme limiting dilution assays were conducted to assess the ability of cells to form colonies, demonstrating a decrease in neurosphere frequency upon LHX2 and EOMES regulation in Group 3 or Group 3/4 cells. HTB-185 and HTB-187 cells were seeded in 24-well ultra-low attachment plates at various concentrations, including 100, 50, 25, 10, and 5 cells per well. Each sphere-forming well was counted and the dilution ratio was plotted based on the number of diluted cells and the number of sphere-forming wells. *P <0.05,****P < 0.0001, ns. P>0.05.

Reference

[1] Northcott, P., Shih, D., Peacock, J. et al. Subgroup-specific structural variation across 1,000 medulloblastoma genomes. Nature 488, 49–56 (2012).
